# Efficiency in Carbon Dioxide Fixation into Cyclic Carbonates: Operating Bifunctional Polyhydroxylated Pyridinium Organocatalysts in Segmented Flow Conditions

**DOI:** 10.3390/molecules28041530

**Published:** 2023-02-04

**Authors:** Lorenzo Poletti, Caterina Rovegno, Graziano Di Carmine, Filippo Vacchi, Daniele Ragno, Arianna Brandolese, Alessandro Massi, Paolo Dambruoso

**Affiliations:** 1 Department of Chemical, Pharmaceutical and Agricultural Sciences, University of Ferrara, Via L. Borsari, 46, 44121 Ferrara, Italy; 2 Institute for Organic Synthesis and Photoreactivity of the Italian National Research Council, CNR Area della Ricerca di Bologna, Via P. Gobetti 101, 40129 Bologna, Italy

**Keywords:** flow chemistry, organocatalysis, carbon dioxide, cyclic carbonates, mass transfer

## Abstract

Novel polyhydroxylated ammonium, imidazolium, and pyridinium salt organocatalysts were prepared through N-alkylation sequences using glycidol as the key precursor. The most active pyridinium iodide catalyst effectively promoted the carbonation of a set of terminal epoxides (80 to >95% yields) at a low catalyst loading (5 mol%), ambient pressure of CO_2_, and moderate temperature (75 °C) in batch operations, also demonstrating high recyclability and simple downstream separation from the reaction mixture. Moving from batch to segmented flow conditions with the operation of thermostated (75 °C) and pressurized (8.5 atm) home-made reactors significantly reduced the process time (from hours to seconds), increasing the process productivity up to 20.1 mmol_(product)_ h^−1^ mmol_(cat)_^−1^, a value ~17 times higher than that in batch mode.

## 1. Introduction

Over the past two decades, there has been a dramatic increase in the amount of CO_2_ being emitted into the atmosphere, resulting in global warming and subsequent environmental harm [1]. Thus, the balance between anthropogenic emissions and removals from the atmosphere is, today, actively pursued to achieve carbon neutrality in the near future [2]. Accordingly, many kinds of liquid and solid sorbents are being investigated for the development of efficient carbon capture and storage (CCS) strategies [3]. In this scenario, reusing CO_2_ as a renewable C1 building block to produce added-value chemicals and fuels is becoming a crucial goal for competitiveness, as CO_2_ refinery may compensate the costs and energy consumption associated with its capture and transportation [4]. However, despite the great interest of academia, industry, and policy makers in carbon capture and utilization (CCU) methodologies, important challenges still need to be addressed including the achievement of levels of process efficiency comparable to those of the petrochemical industry [5]. The kinetic and thermodynamic stability of CO_2_ (intrinsic factor) is the major limitation of CO_2_ utilization as a chemical feedstock, which can be overcome by reacting high-energy substrates such as epoxides and aziridines in the presence of extremely active catalysts. The chemical efficiency of CO_2_ fixation, however, should also take into consideration extrinsic energetic, environmental, and economic factors, thus making preferable the application of inexpensive sustainable catalysts (organocatalysts [6,7] and non-noble metals [8]) with high recyclability, and the operation at a moderate temperature and pressure of CO_2_ (<100 °C, <10 atm) [9,10,11].

Flow chemistry has recently been proven to have great potential as an enabling technology for the process intensification of gas–liquid reactions of CO_2_ [12,13,14,15,16,17,18,19,20,21,22,23,24,25,26,27,28,29,30,31]. In this reaction set-up, millimeter-sized droplets are generated as confined units with increased superficial area determining an improved mass transfer rate across the gas–liquid interface, which is often the rate-limiting step of gaseous CO_2_ reactions. Aside from enhanced kinetics, additional advantages of flow conditions in CCU strategies are the better heat transfer, safety, and process reliability, easy control of pressure, facile scaling-out by extending the period of product collection, and straightforward scale-up using the numbering-up approach [32,33].

**Figure 1:**
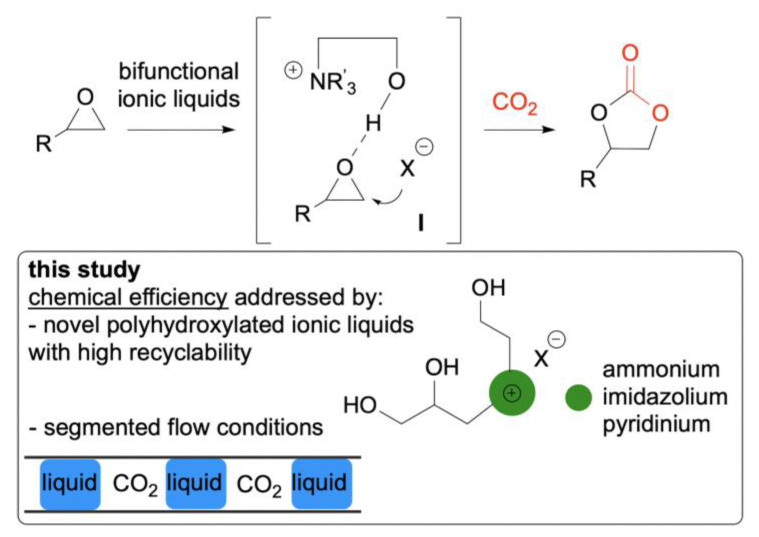
Design of the organocatalytic conversion of CO_2_ into cyclic carbonates under segmented flow conditions.

The atom economical insertion of CO_2_ into epoxides to yield cyclic carbonates is emerging as a strategic transformation for the chemical process industry because it has been estimated that it will consume, together with the dry reforming of methane, up to 25% of waste CO_2_ produced annually [6]. Cyclic carbonates are used as high boiling point aprotic solvents [34,35] and electrolytes in secondary batteries [36,37]; moreover, they are valuable monomers for the production of polycarbonates and polyurethanes [38,39,40,41,42], and intermediates for the synthesis of fine chemicals and pharmaceuticals [43,44]. A plethora of homogeneous and heterogeneous catalytic systems have been proposed for this transformation including metal complexes, metal oxides, organocatalysts, and simple alkaline salts [6,7,8,45,46,47,48,49]; however, only a restricted number of these catalysts can be applied without high temperature and pressure requirements, and be recycled using economical downstream purification steps [9,10,11]. In light of this, various metal-based ionic liquids (ILs) have been recently introduced in the literature, [50,51,52,53] showing good to excellent yields and selectivity, typically by the application of high CO_2_ pressures (up to 50 bar). Additionally, ILs based on quaternary ammonium, imidazolium, and pyridinium salts, eventually immobilized on inorganic [54] and polymeric [55] solid supports, or prepared as hybrid materials, [56] have received considerable attention, showing advantageous features such as uninflammability, low volatility, thermal stability, and flexible structure-tailorability [57,58,59,60,61,62,63,64]. In particular, it has been demonstrated that the presence of hydroxyl groups on the IL moiety significantly increases the catalyst activity as a result of the synergistic effect of hydrogen bonding with the oxygen atom of epoxides, which effectively contributes to the ring-opening process promoted by the halide nucleophilic attack (Figure 1) [65,66,67,68,69,70,71]. On the basis of the same mechanistic rationale, we propose herein a set of novel polyhydroxylated ionic liquids, namely ammonium, imidazolium, and pyridinium organocatalysts, whose activity and recyclability has been initially tested in the carbonation of terminal epoxides under conventional batch conditions. Afterwards, following our interest in the development of efficient technology platforms for the intensification of gas–liquid reactions (aerosol reactors), [72,73] we further developed our study by moving from batch to segmented flow processes with the detection of significant improvements in terms of reaction time and productivity.

## 2. Results and Discussion

**Scheme 1:**
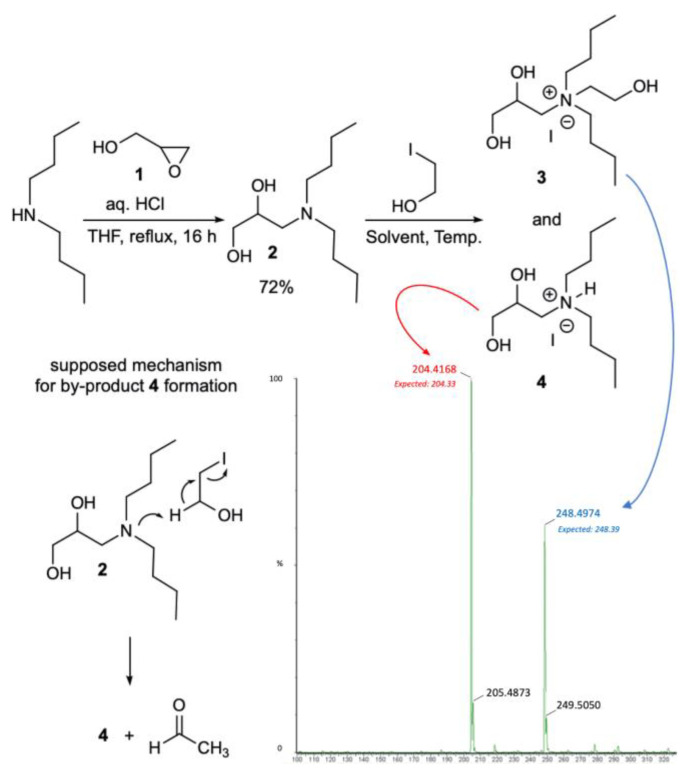
Synthesis of polyhydroxylated ammonium iodide salt **3** and side-reaction path. Solvent and temperature variations are reported in Table 1.

The synthesis of the polyhydroxylated ammonium iodide salt **3** was first addressed by coupling dibutyl amine with glycidol **1** refluxing under acidic conditions to give the intermediate amine **2** in satisfactory yield (72%; Scheme 1). Afterwards, following standard procedures for the N-alkylation of tertiary amines, the diol **2** was refluxed in alcoholic solvent (EtOH) with iodoethanol, affording an inseparable mixture of the target salt **3** (32%) and the by-product **4** (68%), as detected using ^1^H NMR spectroscopy and further confirmed with MS analysis (Table 1, entry 1). Lowering the temperature to 60 °C improved the reaction selectivity toward **3** at the expense, however, of the efficiency conversion (21%; entry 2). Similar unsatisfactory results were also registered using different polar aprotic solvents (DMF, THF) and temperatures (entries 4–5). The unexpected reaction outcome was explained by hydriodic acid elimination from iodoethanol promoted by **2** yielding **4** and acetaldehyde (Scheme 1). Fortunately, we observed that neat conditions (75 °C, 48 h) could suppress the side-reaction path, affording the desired polyhydroxylated ammonium salt **3** as the sole product in quantitative yield and 95% purity (^1^H NMR analysis; entry 8).

**Scheme 2:**
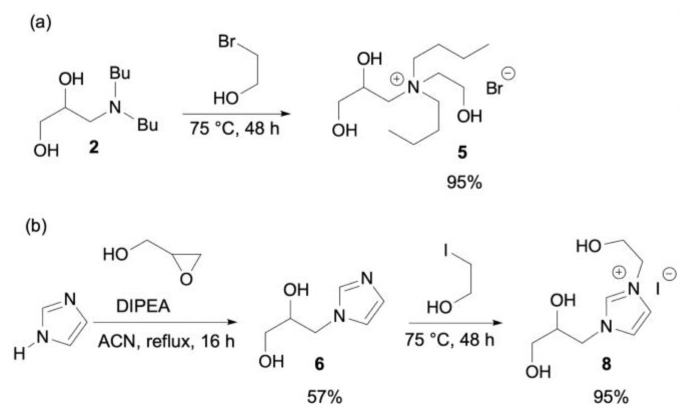
Synthesis of polyhydroxylated (**a**) ammonium- and (**b**) imidazolium-based organocatalysts **5** and **8**.

Next, with the aim of verifying the effect of halide variation on the efficiency of epoxide ring-opening (vide infra), the optimized double N-alkylation sequence was applied to the synthesis of the polyhydroxylated ammonium bromide salt **5** using bromoethanol for quaternarization (Scheme 2). The same protocol was also employed with little modification to produce the bifunctional organocatalyst **8** featuring the designed polyhydroxylated imidazolium moiety.

**Scheme 3:**
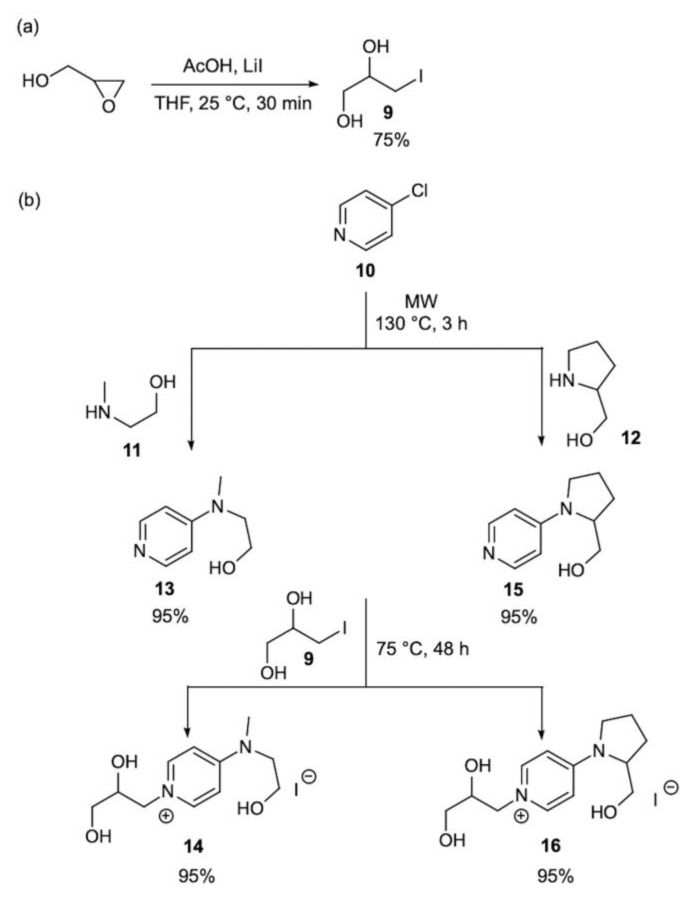
Synthesis of: (**a**) 3-iodopropane-1,2-diol **9** and (**b**) polyhydroxylated pyridinium-based organocatalysts **14** and **16**.

The access to the target pyridinium salts **14** and **16** (Scheme 3) initially required the set-up of a practical procedure for the synthesis of the alkylating agent **9** starting from glycidol, which was identified in this study as the common precursor for introducing the 1,2-propanediol group in the final organocatalysts. The iodide **9** is a known compound and it is typically produced in three steps from glycerol [74]; on the basis of previous observations [75], the straightforward and regioselective conversion of glycidol into the vicinal halohydrin **9** was achieved in satisfactory yield (75%) using lithium iodide in combination with acetic acid under mild reaction conditions. Our synthetic plan proceeded with the synthesis of the intermediate N-alkyl-4-amine pyridines **13** and **15** featuring the hydroxyalkyl chain with a different degree of free rotation. Gratifyingly, microwave-heating (130 °C, 3 h) of the mixtures of 4-chloropyridine **10** with excess of either methylamino-ethanol **11** or prolinol **12** gave pyridines **13** and **15**, respectively, in almost quantitative yields after simple evaporation of unreacted **11**/**12**. Finally, the completion of the synthetic sequence from **13**/**15** was straightforward, affording the polyhydroxylated pyridinium salts **14** and **16** by N-alkylation with iodide **9** under the previously optimized neat conditions.

The catalytic activity of the novel bifunctional organocatalysts **3**, **5**, **8**, **14,** and **16** (10 mol%) was tested at ambient temperature and pressure in the model conversion of styrene oxide **17a** into styrene carbonate **18a** (Table 2). In agreement with the order of nucleophilicity of halide anions and their coordination ability of an acidic hydrogen (intermediate **I**, Figure 1) [6], iodide ammonium salt **3** outperformed the bromide counterpart **5** affording **18a** in 30% yield with complete selectivity (entries 1–2). Among the iodide salts **3**, **8**, **14,** and **16**, the polyhydroxylated pyridinium organocatalyst **16** emerged as the most effective promoter (**18a**: 44%; entry 5), somehow substantiating the importance of some rigidity in the hydroxyalkyl chain for transition state stabilization (**16** vs. **14**).

Different conditions were then screened with the selected organocatalyst **16** to improve the process productivity (Table 3). Increasing the temperature up to 75 °C allowed the full conversion of **17a** with complete selectivity towards **18a** (**16**: 10 mol%; reaction time: 16 h; entries 1–3). Satisfyingly, the same reaction outcome was reproduced by halving the catalyst loading to 5 mol% (entry 4), while a further decrease in the **16** amount (2 mol%) or a shorter reaction time (12 h) resulted in significantly lower conversions (entries 5–6). Based on previous findings [23,70], DMF and H_2_O were tested as additives, detecting, however, a marked drop in reaction efficiency (entries 7–8). It is important to emphasize that under the optimized conditions of entry 4, the reaction mixture at the initial time is heterogeneous, becoming completely homogeneous as the reaction progresses. Therefore, keeping in mind the ultimate goal of process intensification by the application of flow conditions, EtOH (50 mol%) was utilized to obtain full solubilization of catalyst **16** (entry 9); opportunely, only a minimal decrease in the yield of cyclic carbonate **18a** (92%) was observed. Overall, the resultant productivity (*P*, which also corresponds to TOF, Equation (1)) of the optimal batch process (entry 4) was 1.2 mmol_(**18a**)_ h^−1^ mmol_(**16**)_^−1^.

(1) $$P=\frac{\;mol{\;}_{\left(product\right)}}{time{\;}^{.}\;mol{\;}_{\left(catalyst\right)}{}_{\;}}$$

**Figure 2:**
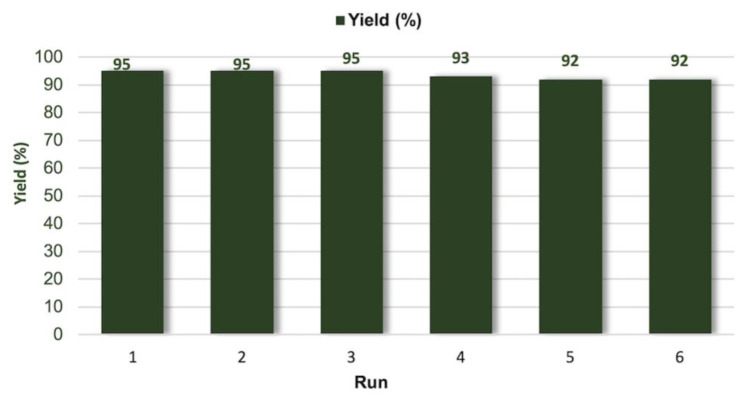
Recycling experiments (optimized conditions of entry 4, Table 3).

The recyclability of the polyhydroxylated pyridinium **16** was investigated over six runs (Figure 2). Upon reaction completion, catalyst recovery consisted of the simple addition of EtOAc. Operating in this way, the catalyst precipitated and the product was collected upon centrifugation. Gratifyingly, only a moderate conversion decrease (~3%) was observed after the fifth recycle, mainly because of the partial loss of catalyst during the recovery and washing steps.

**Scheme 4:**
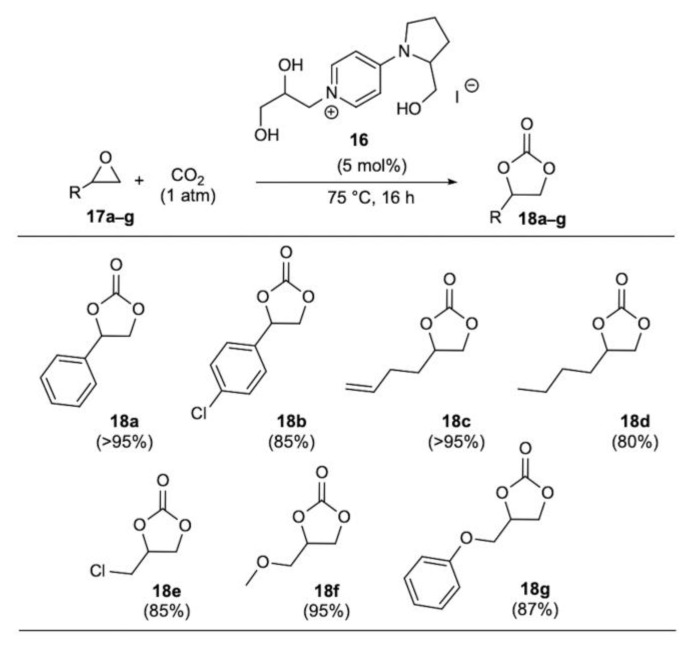
Substrate scope (batch procedure).

The generality and efficacy of the method was tested through a brief substrate scope study, which was conducted with terminal epoxides **17a**–**g** at atmospheric pressure and mild temperature (Scheme 4). In addition to the styrene oxide derivates **17a,b**, the epoxides displaying an alkyl chain **17c**–**g** could also be converted into the corresponding cyclic carbonates **18a**–**g** in good to excellent yields (80% to >95%).

**Scheme 5:**
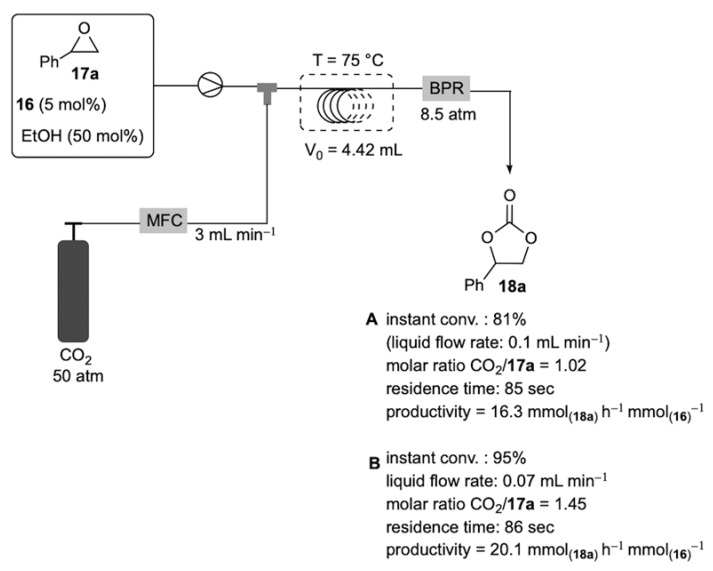
Production of styrene carbonate **18a** under segmented flow conditions.

At this stage of the study, driven by our interest in process intensification by the application of flow techniques [76,77,78,79,80,81,82,83,84,85,86,87,88], we next investigated the transition of the model carbonation of styrene oxide from batch to segmented flow conditions [89,90]. The *in-house* assembled flow apparatus consisted of a 4.42 mL spiral capillary reactor (FEP tubing; 0.75 mm ID) placed inside a thermostated bath (75 °C). The coil was connected to an HPLC pump and a CO_2_ cylinder by means of a standard T junction, where the gas and liquid streams were mixed. The exact CO_2_ volume was delivered into the reactor by a mass flow controller (MFC), while a back-pressure regulator (BPR) maintained a constant pressure of CO_2_ (8.5 atm) throughout the system (Scheme 5 and Supplementary Material Figure S1). Initial experiments were performed to identify a stable segmented flow regime by variation of the liquid and gas flow rates, always keeping a molar excess of CO_2_ over styrene oxide. Approximately, each segment length was found to be in a range of 0.5 to 1.0 mm. The residence time (t_r_) was calculated as the ratio of reactor volume over the total gas and liquid flow rate. After some experimentation, two set of conditions (**A** and **B**) were optimized with a constant CO_2_ flow rate of 3.00 mL min^−1^ and liquid flow rates of 0.10 and 0.07 mL min^−1^ corresponding to CO_2_/**17a** molar ratios of 1.02 and 1.45, respectively. Significantly, after the attainment of the steady-state regime (ca. 2 min), condition **A** provided **18a** with an instant conversion of 81% (^1^H NMR of the outlet stream) in only 85 s of residence time, thus resulting in a process productivity of 16.3 mmol_(**18a**)_ h^−1^ mmol_(**16**)_^−1^. In accordance with our expectations, the reduction in the liquid flow rate to 0.07 mL min^−1^ (condition **B**) further increased the reaction conversion (>95%) with almost the same residence time (86 s), affording **18a** with a productivity of 20.1 mmol_(**18a**)_ h^−1^ mmol_(**16**)_^−1^. This value is about 17-fold higher than that measured in batch-mode and it was explained by the improved mass transfer of CO_2_ at the gas–liquid interphase due to the increased pressure and segmented flow regime.

The continuous production of cyclic carbonates **18b**–**g** was finally examined under the optimized flow conditions, affording remarkable conversion efficiencies (>85%) and productivities in the range of 17.2–20.1 mmol_(**18**)_ h^−1^ mmol_(**16**)_^−1^ (Table 4).

## 3. Materials and Methods

Commercially available reagents were purchased from commercial sources and used without any subsequent purification. The solvents used for reactions were distilled from appropriate drying agents and stored over 3 Å molecular sieves. ^1^H-NMR and ^13^C-NMR spectra were recorded on Varian Mercury Plus 300 (Varian Inc., Palo Alto, CA, USA) and Varian Mercury Plus 400 (Varian Inc., Palo Alto, CA, USA) spectrometers in CDCl_3_, DMSO-d_6_, and D_2_O at room temperature. ^13^C{^1^H} NMR spectra were recorded in ^1^H broad-band decoupled mode, and chemical shifts (δ) are reported in parts per million relative to the residual solvent peak. Flash column chromatography was performed on silica gel 60 (230−400 mesh). High-resolution mass spectra (HRMS) were recorded in positive ion mode with an Agilent 6520 HPLC-Chip Q/TF-MS nanospray instrument (Agilent Technologies, Santa Clara, CA, USA) using a time-of-flight, a quadrupole, or a hexapole unit to produce spectra.

### 3.1. Procedures for the Synthesis of Intermediates ***2***, ***6***, ***9***, ***13***, ***15*** and Organocatalysts ***3***, ***5***, ***8***, ***14***, ***16***

*3-(Dibutylamino)propane-1,2-diol***(2)**. Glycidol (15.0 mmol) in THF (10 mL), HCl 37% (1 mL), and dibutyl amine (5 mmol) was added to a round-bottom flask with a refrigerator on top. The mixture was stirred and refluxed overnight. Once the dibutyl amine was completely reacted, the product was purified by acid/base extraction with DCM. The reaction crude was solubilized in DCM and then extracted with a solution of HCl 1M; the organic layer was removed and the aqueous phase was basified with NaOH 1M solution and re-extracted with DCM. The organic layer was treated with anhydrous sodium sulphate and filtered, and the solvent was removed using a rotary evaporator and high-vacuum pump. By following this procedure, 3-(dibutylamino)propane-1,2-diol **2** was obtained as a yellow viscous oil (3.60 mmol, 72% yield). ^1^H NMR (500 MHz, CDCl_3_) δ 5.73–5.59 (m, 3H), 5.45 (dd, *J* = 11.1, 4.5 Hz, 1H), 4.59–4.42 (m, 3H), 4.42–4.31 (m, 3H), 3.47–3.33 (m, 4H), 3.33–3.18 (m, 4H), 2.87 (t, *J* = 7.4 Hz, 6H). ^13^C{^1^H} NMR (126 MHz, CDCl_3_): δ 67.3, 65.0, 57.0, 54.3, 29.2, 20.7, 14.1. HRMS (ESI) *m/z*: [M + H]^+^ calcd for C_11_H_26_NO_2_^+^ 204.1958, found 204.1952.

*N-Butyl-N-(2,3-dihydroxypropyl)-N-(3-hydroxypropyl)butan-1-aminium iodide* **(3)**. Compound **2** (2.50 mmol) and 2-iodoethanol (2.50 mmol) were added to a 10 mL vial equipped with a small magnetic bar. The vial was sealed, and an argon atmosphere inside the reaction vial was created by three cycles of vacuum and argon pumping. The mixture was vigorously stirred for 48 h at 75 °C to obtain the desired product. No additional purification step is needed. By following this procedure, *N*-butyl-*N*-(2,3-dihydropropyl)-*N*-(3-hydroxypropyl)butan-1-aminium iodide **(3)** was obtained as a very viscous brown liquid (2.50 mmol, quant.) with a purity of ca. 95% (^1^H NMR analysis). ^1^H NMR (300 MHz, DMSO-d_6_) δ 5.31 (d, *J* = 5.3 Hz, 1H), 5.20 (t, *J* = 5.3 Hz, 1H), 5.04 (t, *J* = 5.3 Hz, 1H), 4.03–3.88 (m, 1H), 3.87–3.71 (m, 2H), 3.63–3.55 (m, 1H), 3.50–3.35 (m, 5H), 3.32–3.11 (m, 3H), 3.01 (broad s, 1H), 1.78–1.46 (m, 4H), 1.36–1.17 (m, 4H), 0.95–0.83 (m, 6H). ^13^C{^1^H} NMR (101 MHz, DMSO-d_6_) δ 66.0, 64.1, 62.8, 61.8, 60.6, 59.8, 59.8, 56.5, 55.0, 23.6, 19.6, 19.0, 13.9. HRMS (ESI) *m/z*: [M]^+^ calcd for C_13_H_30_NO_3_^+^ 248.2226 found 248.2232.

*N-Butyl-N-(2,3-dihydroxypropyl)-N-(3-hydroxypropyl)butan-1-aminium bromide* **(5)**. Compound **2** (2.50 mmol) and 2-bromoethanol (2.50 mmol) were added to a 10 mL vial equipped with a small magnetic bar. The vial was sealed, and an argon atmosphere inside the reaction vial was created by three cycles of vacuum and argon pumping. The mixture was vigorously stirred for 48 h at 75 °C to obtain the desired product. No purification steps are needed. By following this procedure, *N*-butyl-*N*-(2,3-dihydroxypropyl)-*N*-(3-hydroxypropyl)butan-1-aminium bromide **(5)** was obtained as a very viscous brown liquid (2.50 mmol, quant.) with a purity of ca. 95% (^1^H NMR analysis). ^1^H NMR (300 MHz, DMSO-d_6_) δ 5.33 (d, *J* = 5.2 Hz, 1H), 5.21 (t, *J* = 5.2 Hz, 1H), 5.04 (t, *J* = 5.2 Hz, 1H), 4.04–3.90 (m, 1H), 3.89–3.73 (m, 2H), 3.73–3.52 (m, 1H), 3.50–3.34 (m, 6H), 3.31–3.12 (m, 2H), 3.03 (s, 1H), 1.81–1.46 (m, 4H), 1.38–1.16 (m, 4H), 0.96–0.81 (m, 6H).^13^C{^1^H} NMR (101 MHz, DMSO-d_6_) δ 66.0, 64.1, 62.7, 61.8, 60.6, 59.8, 59.8, 56.5, 55.0, 23.6, 19.6, 19.0, 13.9. HRMS (ESI) *m/z*: [M]^+^ calcd for C_13_H_30_NO_3_^+^ 248.2226 found 248.2229.

*3-(1H-Imidazol-1-yl)propane-1,2-diol* **(6)**. Glycidol (15.0 mmol) in acetonitrile (10 mL), DIPEA (10 mmol), and imidazole (10 mmol) were added to a round-bottom flask with a refrigerator on top. The mixture was stirred and refluxed overnight. Once the reaction was complete, the purification took place through flash chromatography using an automatic flash chromatographer CombiFlash. The elution gradient was from 100% A to 100% B in 45 CV, then 100% B in 10 CV (A: AcOEt + 2% NH_4_OH, B: AcOEt/MeOH = 9/1 + 2% NH_4_OH). By following this procedure, 3-(1H-imidazol-1-yl)propane-1,2-diol **(6)** was obtained as a pale yellow viscous oil (5.70 mmol, 57% yield). ^1^H NMR (500 MHz, D_2_O) δ 7.68 (s, 1H), 7.18 (s, 1H), 7.03 (s, 1H), 4.19 (dd, *J* = 13.7, 3.0 Hz, 1H), 4.06–3.98 (m, 2H), 3.60 (dd, *J* = 11.8, 5.0 Hz, 1H), 3.53 (dd, *J* = 11.8, 5.0 Hz, 1H).^13^C{^1^H} NMR (126 MHz, D_2_O): δ 138.2, δ 127.2, 120.5, 70.6, δ 62.4, δ 49.0. HRMS (ESI) *m/z*: [M + H]^+^ calcd for C_6_H_11_N_2_O_2_^+^ 143.0815 found 143.0816.

*1-(2,3-Dihydroxypropyl)-3-(2-hydroxyethyl)-1H-imidazol-3-ium iodide* **(8)**. Compound **6** (1.40 mmol) and 2-iodoethanol (1.40 mmol) were added to a 10 mL vial equipped with a small magnetic stir bar. The vial was hermetically sealed, and an argon atmosphere inside the reaction vial was created by three cycles of vacuum and argon pumping. The mixture was stirred for 24 h at 75 °C to give the final product. No purification steps are needed. By following this procedure, 1-(2,3-dihydroxypropyl)-3-(2-hydroxyethyl)-1H-imidazol-3-ium iodide **(8)** was obtained as a very viscous brown liquid (1.40 mmol, quant.) with a purity of ca. 95% (^1^H NMR analysis). ^1^H NMR (300 MHz, DMSO-d_6_) δ 9.05 (s, 1H), 7.69 (d, *J* = 9.4 Hz, 2H), 5.29 (d, *J* = 5.0 Hz, 1H), 5.14 (t, *J* = 5.0 Hz, 1H), 4.93 (t, *J* = 5.0 Hz, 1H), 4.30 (dd, *J* = 13.8, 2.7 Hz, 1H), 4.21 (t, *J* = 5.0 Hz, 2H), 4.07 (dd, *J* = 13.8, 8.2 Hz, 1H), 3.83–3.68 (m, 3H), 3.46–3.36 (m, 1H), 3.26–3.17 (m, 1H). ^13^C{^1^H} NMR (101 MHz, DMSO-d_6_) δ 137.2, 123.5, 122.8, 70.1, 63.2, 59.8, 52.6, 52.0. HRMS (ESI) *m/z*: [M]^+^ calcd for C_8_H_15_N_2_O_3_^+^ 187.1077 found 187.1071.

*3-Iodopropane-1,2-diol* **(9)**. LiI (80.0 mmol, 10 g) was added to a solution of glycidol (50.0 mmol) and acetic acid (150 mmol) in anhydrous THF (40 mL), and the solution was kept at room temperature and stirred in argon atmosphere for 40 min. The mixture was diluted with distilled water and extracted with two aliquots of ethyl acetate (2 × 20 mL). The organic layer was treated with anhydrous sodium sulphate and filtered, and the solvent was removed using a rotary evaporator and high-vacuum pump. By following this procedure, 3-iodopropane-1,2-diol **(9)** was obtained as a yellow amorphous solid (50.0 mmol, quant.) ^1^H NMR (400 MHz, D_2_O) δ 3.59–3.42 (m, 3H), 3.25 (dd, *J* = 10.8, 4.5 Hz, 1H), 3.15 (dd, *J* = 10.8, 4.5 Hz, 1H). ^13^C{^1^H} NMR (101 MHz, D_2_O) δ 70.6, 64.4, 8.6. HRMS (ESI) *m/z*: [M + H]^+^ calcd for C_3_H_8_IO_2_^+^ 202.9563 found 202.9554 [74].

*2-(Methyl(pyridin-4-yl)amino)ethan-1-ol* **(13)**. Chloropyridine hydrochloride (5.00 mmol) and *N*-methylethanolamine (62.5 mmol) were added to a round-bottom flask. The mixture was stirred at 120°C for 24 h. Once the reaction was complete, the excess of unreacted amine was vacuum-evaporated, and then the product was purified by extracting the free amine with DCM from a basic environment (K_2_CO_3_). The organic layer was treated with anhydrous sodium sulphate and filtered, and the solvent was removed using a rotary evaporator and high-vacuum pump. By following this procedure, 2-(methyl(pyridin-4-yl)amino)ethan-1-ol **(13)** was obtained as a white amorphous solid (3.50 mmol, 70% yield). ^1^H NMR (400 MHz, CDCl_3_) δ 8.12 (dd, *J* = 5.1, 1.6 Hz, 2H), 6.52 (dd, *J* = 5.1, 1.6 Hz, 2H), 3.83 (t, *J* = 5.7 Hz, 2H), 3.54 (t, *J* = 5.7 Hz, 2H), 3.04 (s, 3H), 2.70 (broad s, 1H).^13^C{^1^H} NMR (126 MHz, CDCl_3_): δ 149.0, 106.7, 59.6, 53.6, 38.2. HRMS (ESI) *m/z*: [M + H]^+^ calcd for C_8_H_13_N_2_O^+^ 153.1022 found 153.1024.

*1-(2,3-Dihydroxypropyl)-4-((2-hydroxyethyl)(methyl)amino)pyridin-1-ium iodide* **(14)**. Compound **13** (0.25 mmol) and 3-iodopropane-1,2-diol **9** (0.25 mmol) were added to a 10 mL vial equipped with a small magnetic bar. The vial was hermetically sealed, and an argon atmosphere inside the reaction vial was created by three cycles of vacuum and argon pumping. The mixture was stirred for 24 h at 75 °C to give the final product. No purification steps are needed. By following this procedure, 1-(2,3-dihydroxypropyl)-4-((2-hydroxyethyl)(methyl)amino)pyridin-1-ium iodide **(14)** was obtained as a very viscous yellow liquid (0.25 mmol, quant.) with a purity of ca. 95% (^1^H NMR analysis). ^1^H NMR (300 MHz, DMSO-d_6_) δ 8.13 (dd, *J* = 12.5, 7.5 Hz, 2H), 7.12 (d, *J* = 5.2 Hz, 1H), 6.96 (d, *J* = 7.5 Hz, 1H), 5.24 (d, *J* = 5.2 Hz, 1H), 4.92 (dd, *J* = 12.0, 6.2 Hz, 2H), 4.27 (d, *J* = 10.6 Hz, 1H), 4.01 (dd, *J* = 13.6, 8.2 Hz, 1H), 3.73 (broad s, 1H), 3.63 (s, 4H), 3.53–3.40 (m, 2H), 3.15 (s, 3H). ^13^C{^1^H} NMR (101 MHz, DMSO-d_6_) δ 156.6, 143.5, 142.8, 108.0, 107.6, 70.6, 63.1, 60.0, 58.4, 54.2. HRMS (ESI) *m/z*: [M]^+^ calcd for C_11_H_19_N_2_O_3_^+^ 227.1390 found 227.1380.

*(1-(Pyridin-4-yl)pyrrolidin-2-yl)methanol* **(15)**. 4-Chloropyridine hydrochloride (0.70 mmol) and prolinol (8.75 mmol) were added to a 5 mL vial equipped with a magnetic stir bar. The vial was hermetically sealed and inserted into a microwave. The reaction mixture was stirred and irradiated for 3 h at 120 °C. After this time, the crude was transferred into a round-bottom flask and attached to a high-vacuum pump in order to remove the unreacted excess of prolinol. After 24 h, the crude was diluted in dichloromethane and washed with a 1M solution of K_2_CO_3_. The organic layer was concentrated at the rotary evaporator and the flask was attached to the high-vacuum pump for an additional 24 h. By following this procedure, (1-(pyridine-4-yl)pyrrolidine-2-yl)methanol **(15)** was obtained as a brown amorphous solid (0.70 mmol, quant.). ^1^H NMR (300 MHz, CDCl_3_) δ 8.16 (d, *J* = 5.0 Hz, 2H), 6.48 (d, *J* = 5.0 Hz, 2H), 3.98–3.83 (m, 1H), 3.70 (dd, *J* = 11.0, 4.2 Hz, 1H), 3.60 (dd, *J* = 11.0, 6.9 Hz, 1H), 3.48–3.43 (m, 1H), 3.28–3.08 (m, 1H), 2.20–1.90 (m, 6H). ^13^C{^1^H} NMR (101 MHz, CDCl_3_) δ 149.6, 107.4, 62.6, 59.6, 48.1, 28.3, 23.2. HRMS (ESI) *m/z*: [M + H]^+^ calcd for C_10_H_15_N_2_O^+^ 179.1179 found 179.1181.

*1-(2,3-Dihydroxypropyl)-4-(2-(hydroxymethyl)pyrrolidin-1-yl)pyridin-1-ium iodide* **(16)**. Compound **15** (0.25 mmol) and 3-iodopropane-1,2-diol **9** (0.25 mmol) was added to a 10 mL vial equipped with a small magnetic stir bar. The vial was hermetically sealed, and an argon atmosphere inside the reaction vial was created by three cycles of vacuum and argon pumping. The mixture was stirred for 24 h at 75 °C. No purification steps are needed. By following this procedure, 1-(2,3-dihydroxypropyl)-4-(2-(hydroxymethyl)pyrrolidin-1-yl)pyridin-1-ium iodide **(16)** was obtained as a very viscous brown liquid (0.25 mmol, quant.) with a purity of ca. 95% (^1^H NMR analysis). ^1^H NMR (300 MHz, DMSO-d_6_) δ 8.16 (t, *J* = 6.3 Hz, 2H), 7.06 (dd, *J* = 7.8, 2.8 Hz, 1H), 6.86 (dd, *J* = 7.8, 2.8 Hz, 1H), 5.25 (d, *J* = 5.4 Hz, 1H), 4.96 (dt, *J* = 13.1, 5.4 Hz, 2H), 4.29 (d, *J* = 12.3 Hz, 1H), 4.14 (d, *J* = 5.1 Hz, 1H), 4.03 (dd, *J* = 13.6, 7.8 Hz, 1H), 3.75 (broad s, 1H), 3.58 (t, *J* = 10.8 Hz, 1H), 3.51–3.35 (m, 4H), 3.31–3.21 (m, 1H), 2.23–1.71 (m, 4H). ^13^C{^1^H} NMR (101 MHz, DMSO-d_6_) δ 154.0, 143.5, 142.9, 108.8, 108.4, 70.8, 63.2, 61.3, 61.1, 60.1, 49.2, 28.0, 22.8. HRMS (ESI) *m/z*: [M]^+^ calcd for C_13_H_21_N_2_O_2_^+^ 253.1547 found 253.1551.

### 3.2. General Procedure for the Synthesis of Styrene Carbonates ***18a***–***g*** under Batch Conditions

Epoxide **17** (2.00 mmol) and catalyst **16** (5 mol%) were added to a 10 mL vial equipped with a small magnetic stir bar. A CO_2_ atmosphere inside the reaction vial was created by three cycles of vacuum and CO_2_ pumping and maintained by a carbon dioxide balloon connected via a needle. The mixture was stirred for 16 h at room temperature, then diluted with EtOAc to precipitate the catalyst **16**, and centrifuged to recover the cyclic carbonate **18** in the solution, which was purified using column chromatography.

*4-Phenyl-1,3-dioxolan-2-one* **(18a)**. By following the general procedure, **18a** (318 mg, 1.94 mmol, >95%) was obtained as a viscous colorless oil after short column chromatography on silica gel (9:1 cyclohexane/EtOAc). ^1^H NMR (500 MHz, CDCl_3_) δ 7.48–7.41 (m, 3H), 7.36 (dd, *J* = 7.8, 1.8 Hz, 2H), 5.67 (t, *J* = 8.0 Hz, 1H), 4.80 (t, *J* = 8.0 Hz, 1H), 4.34 (t, *J* = 8.0 Hz, 1H). ^13^C{^1^H} NMR (126 MHz, CDCl_3_) δ 154.8, 135.8, 129.7, 129.2, 125.9, 78.0, 71.2. HRMS (ESI) *m/z*: [M + H]^+^ calcd for C_9_H_9_O_3_^+^ 165.0546 found 165.0539 [23].

*4-(4-Chlorophenyl)-1,3-dioxolan-2-one* **(18b)**. By following the general procedure, **18b** (356 mg, 1.70 mmol, 85%) was obtained as a viscous pale yellow oil after column chromatography on silica gel (9:1 cyclohexane/EtOAc). ^1^H NMR (300 MHz, CDCl_3_) δ 7.43 (d, *J* = 8.5 Hz, 2H), 7.31 (d, *J* = 8.5 Hz, 2H), 5.66 (t, *J* = 8.0 Hz, 1H), 4.80 (t, *J* = 8.6 Hz, 1H), 4.31 (dd, *J* = 8.6, 8.0 Hz, 1H). ^13^C{^1^H} NMR (101 MHz, CDCl_3_) δ 154.5, 135.8, 134.2, 129.5, 127.2, 77.0, 71.0. HRMS (ESI) *m/z*: [M + H]^+^ calcd for C_9_H_8_ClO_3_^+^ 199.0156 found 199.0163 [91].

*4-(But-3-en-1-yl)-1,3-dioxolan-2-one* **(18c)**. By following the general procedure, **18c** (276 mg, 1.95 mmol, >95%) was obtained as a viscous colorless oil after short column chromatography on silica gel (9:1 cyclohexane/EtOAc). ^1^H NMR (400 MHz, CDCl_3_) δ 5.88–5.70 (m, 1H), 5.15–5.01 (m, 2H), 4.78–4.67 (m, 1H), 4.53 (t, *J* = 8.2 Hz, 1H), 4.08 (t, *J* = 8.2 Hz, 1H), 2.34–2.10 (m, 2H), 2.00–1.87 (m, 1H), 1.83–1.71 (m, 1H). ^13^C{^1^H} NMR (101 MHz, CDCl_3_) δ 154.9, 136.0, 116.5, 76.3, 69.3, 33.1, 28.7. HRMS (ESI) *m/z*: [M + H]^+^ calcd for C_7_H_11_O_3_^+^ 143.0703 found 143.0699 [23].

*4-Butyl-1,3-dioxolan-2-one* **(18d)**. By following the general procedure, **18d** (230 mg, 1.60 mmol, 80%) was obtained as a viscous colorless oil after column chromatography on silica gel (9:1 cyclohexane/EtOAc). ^1^H NMR (400 MHz, CDCl_3_) δ 4.75–4.65 (m, 1H), 4.52 (t, *J* = 8.1 Hz, 1H), 4.07 (dd, *J* = 8.1, 7.1 Hz, 1H), 1.88–1.76 (m, 1H), 1.74–1.64 (m, 1H), 1.48–1.31 (m, 4H), 0.93 (t, *J* = 7.1 Hz, 3H). ^13^C{^1^H} NMR (101 MHz, CDCl_3_) δ 155.0, 77.0, 69.4, 33.6, 26.4, 22.2, 13.8. HRMS (ESI) *m/z*: [M + H]^+^ calcd for C_7_H_13_O_3_^+^ 145.0859 found 145.0861 [23].

*4-(Chloromethyl)-1,3-dioxolan-2-one* **(18e)**. By following the general procedure, **18e** (232 mg, 1.70 mmol, 85%) was obtained as a viscous colorless oil after column chromatography on silica gel (9:1 cyclohexane/EtOAc). ^1^H NMR (400 MHz, CDCl_3_) δ 4.99–4.91 (m, 1H), 4.59 (dd, *J* = 8.9, 8.2 Hz, 1H), 4.41 (dd, *J* = 8.9, 5.7 Hz, 1H), 3.84–3.68 (m, 2H). ^13^C{^1^H} NMR (101 MHz, CDCl_3_) δ 154.0, 74.2, 67.0, 43.5. HRMS (ESI) *m/z*: [M + H]^+^ calcd for C_4_H_6_ClO_3_^+^ 137.0000 found 136.9998 [23].

*4-(Methoxymethyl)-1,3-dioxolan-2-one* **(18f)**. By following the general procedure, **18f** (250 mg, 1.90 mmol, 95%) was obtained as a viscous colorless oil after short column chromatography on silica gel (9:1 cyclohexane/EtOAc). ^1^H NMR (300 MHz, CDCl_3_) δ 4.85–4.75 (m, 1H), 4.49 (t, *J* = 8.4 Hz, 1H), 4.38 (dd, *J* = 8.4, 6.1 Hz, 1H), 3.61 (qd, *J* = 10.9, 3.9 Hz, 2H), 3.43 (s, 3H). ^13^C{^1^H} NMR (101 MHz, CDCl_3_) δ 154.9, 74.9, 71.4, 66.2, 59.7. HRMS (ESI) *m/z*: [M + H]^+^ calcd for C_4_H_9_O_4_^+^ 133.0495 found 133.0489 [92].

*4-(Phenoxymethyl)-1,3-dioxolan-2-one* **(18g)**. By following general procedure, **18g** (337 mg, 1.74 mmol, 87%) was obtained as a white amorphous solid after trituration in Et_2_O. ^1^H NMR (300 MHz, CDCl_3_) δ 7.31 (dd, *J* = 8.2, 7.3 Hz, 2H), 7.02 (t, *J* = 7.3 Hz, 1H), 6.91 (d, *J* = 8.2 Hz, 2H), 5.09–4.98 (m, 1H), 4.66–4.50 (m, 2H), 4.20 (qd, *J* = 10.5, 3.9 Hz, 2H). ^13^C{^1^H} NMR (101 MHz, CDCl_3_) δ 157.7, 129.7, 122.0, 114.6, 74.0, 66.8, 66.2. HRMS (ESI) *m/z*: [M + H]^+^ calcd for C_10_H_11_O_4_^+^ 195.0652 found 195.0660 [23].

### 3.3. General Procedure for the Synthesis of Cyclic Carbonates ***18a***–***g*** under Segmented Flow Conditions

Epoxide **17**, ethanol (50 mol%), and organocatalysts **16** (5 mol%) were mixed in the reservoir and the resulting solution was pumped through the thermostated reactor (75 °C) at 0.07 mL min^−1^. Simultaneously, a CO_2_ gas flow of 3.0 mL min^−1^ was delivered. Collection and analysis using ^1^H NMR of the outlet stream (minute by minute with durene as internal standard for conversion evaluation) was started 4 min after injection and maintained for an additional 6 min. After this period, the collected reaction mixture was diluted with EtOAc to precipitate the catalyst **16**, and centrifuged to recover the cyclic carbonate **18** in the solution, which was purified as described in the batch procedure (Section 3.2).

## 4. Conclusions

In summary, the chemical efficiency of the carbonation of terminal epoxides with CO_2_ to produce cyclic carbonates has been investigated by reporting a set of novel polyhydroxylated ionic liquids and operating segmented flow reactors. The selected pyridinium iodide organocatalyst guaranteed high conversions at ambient pressure and moderate temperature (75 °C), showing high reusability and simple downstream separation in batch experiments. Transition to segmented flow conditions determined a ∼17-fold increase in process productivity and a reduction in process time from hours to seconds, as a result of the improved CO_2_ mass transfer at the gas–liquid interphase due to the moderate increase in pressure (8.5 atm) and the segmented flow regime. Therefore, we believe that the flow methodology herein disclosed might represent a new opportunity for further advancements in the process intensification of CO_2_ fixation into cyclic carbonates.

## Figures and Tables

**Table 1:** Optimization of ammonium salt **3** synthesis.

Entry ^1^	Solvent	Temp. (°C)	3 (%) ^2^	4 (%) ^2^
1	EtOH	reflux	32	68
2	EtOH	60	21	<5
3	DMF	80	24	76
4	DMF	70	18	27
5	THF	reflux	41	59
6	neat	25	<5	-
7	neat	60	49	-
8	neat	75	>95	-

^1^ Conditions: **2** (2.5 mmol), 2-iodoethanol (2.5 mmol). ^2^ Detected with ^1^H NMR of the crude reaction mixture with durene as internal standard.

**Table 2:** Screening of organocatalyst **3**, **5**, **8**, **14**, and **16** activity.

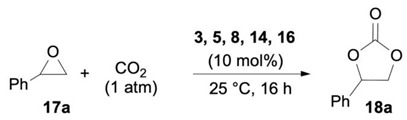				
Entry ^1^	Catalyst	Conv. (%) ^2^	Sel. (%) ^2^	18a (%) ^2^
1	**3**	30	>99	30
2	**5**	5	>99	5
3	**8**	27	>99	27
4	**14**	35	>99	35
5	**16**	44	>99	44

^1^ **17a** (2.00 mmol), CO_2_ (1 atm, balloon), neat conditions. ^2^ Detected with ^1^H NMR of the crude reaction mixture with durene as internal standard.

**Table 3:** Optimization study with catalyst **16** under batch conditions.

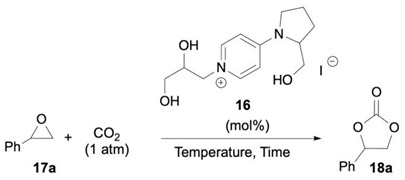						
Entry ^1^	16 (mol%)	Temp. (°C)	Time (h)	Conv. (%) ^2^	Sel. (%) ^2^	18a (%) ^2^
1	10	25	16	44	>99	44
2	10	50	16	62	>99	62
3	10	75	16	>95	>99	95
4	5	75	16	>95	>99	95
5	2	75	16	74	>99	74
6	5	75	12	88	>99	88
7 ^3^	5	75	16	15	>99	15
8 ^4^	5	75	16	48	>99	48
9 ^5^	5	75	16	92	>99	92

^1^ **17a** (2.00 mmol), CO_2_ (1 atm, balloon), neat conditions. ^2^ Detected with ^1^H NMR of the crude reaction mixture with durene as internal standard. ^3^ Additive: DMF (5.00 mmol). ^4^ Additive: H_2_O (50 mol%). ^5^ Additive: EtOH (50 mol%).

**Table 4:** Production of cyclic carbonates **18** under segmented flow conditions.

Entry ^1^	18	Conv. (%) ^2^	*P* ^3^
1	**18a**	>95	20.1
2	**18b**	90	18.0
3	**18c**	>95	20.0
4	**18d**	86	17.2
5	**18e**	91	18.1
6	**18f**	>95	20.0
7	**18f**	92	18.4

^1^ **17**, **16** (5 mol%), EtOH (50 mol%), pressure: 8.5 atm; temperature: 75 °C; residence time: 86 sec; CO_2_ flow rate: 3 mL min^−1^; liquid flow rate: 0.07 mL min^−1^. See Scheme 5 and Figure S1 for the flow apparatus. ^2^ Instant conversion in the steady-state regime as determined with ^1^H NMR analysis. ^3^ Productivities (*P*) are measured in mmol_(**18**)_ h^−1^ mmol_(**16**)_^−1^.

## Data Availability

Not applicable.

## Supplementary Materials

The following supporting information can be downloaded at: https://www.mdpi.com/article/10.3390/molecules28041530/s1. Figure S1: Flow apparatus. Figures S2 and S3: ^1^H and ^13^C NMR spectra of intermediates and organocatalysts (2, 3, 5, 6, 8, 9, 13, 14, 15,16); Figure S12–S18: ^1^H and ^13^C NMR spectra of cyclic carbonates (18a–18g).

## Sample Availability

Samples of the compounds are available from the authors by request.
