# Supplementary material for: Efficiency in Carbon Dioxide Fixation into Cyclic Carbonates: Operating Bifunctional Polyhydroxylated Pyridinium Organocatalysts in Segmented Flow Conditions
Source: Molecules. 2023 Feb 4;28(4):1530. doi: 10.3390/molecules28041530 (PMC9960811; doi:10.3390/molecules28041530)

Supporting materials

Efficiency in Carbon Dioxide Fixation into Cyclic Carbonates: Operating Bifunctional Polyhydroxylated Pyridinium Organocatalysts in Segmented Flow Conditions

Lorenzo Poletti,[a] Caterina Rovegno,[a,b] Graziano Di Carmine,[a] Filippo Vacchi,[b] Daniele Ragno,[a] Arianna Brandolese,[a] Alessandro Massi[a,*] and Paolo Dambruoso[b,*]

[a] Department of Chemical, Pharmaceutical and Agricultural Sciences, University of Ferrara, Via L. Borsari, 46 – 44121 Ferrara (Italy)

[b] Institute for Organic Synthesis and Photoreactivity of the Italian National Research Council, Area della Ricerca di Bologna, Via P. Gobetti, 101 – 40129 – Bologna (Italy)

Table of contents

1. Flow apparatus (Figure S1) S2
2. ^{1}H and ^{13}C NMR spectra of intermediates **2, 6, 9, 13, 15** and organocatalysts **3, 5, 8, 14, 16** S3
3. ^{1}H and ^{13}C NMR spectra of cyclic carbonates **18a-g** S13

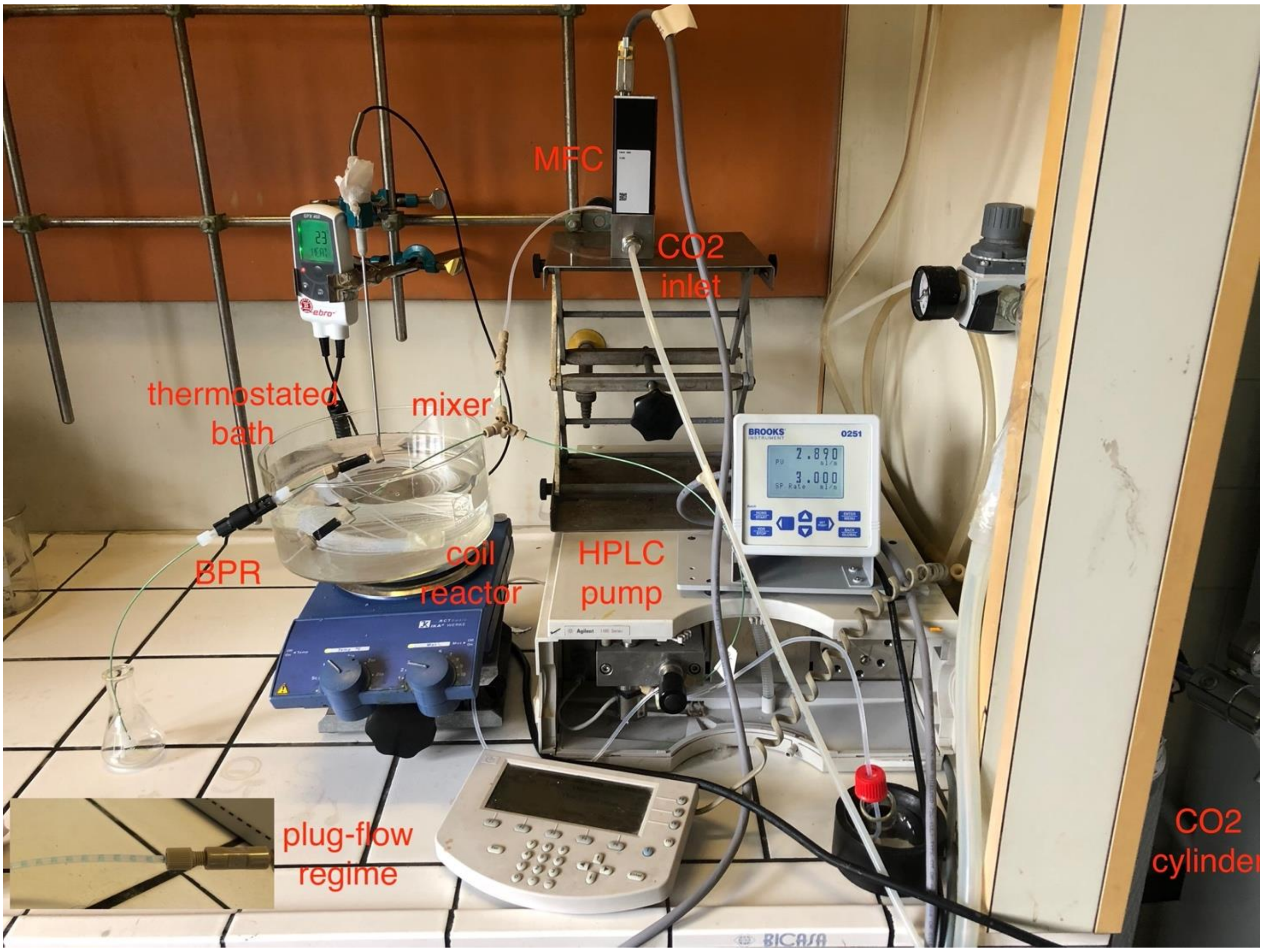

Figure S1. Flow apparatus

The volume of gaseous CO_2 is controlled by the mass flow controller (MFC) and mixed with the liquid phase in a T-mixer. The reactor consists of a 4.42 mL spiral capillary (FEP tubing; 0.75 mm internal diameter; length 10 m) placed inside a thermostated bath. The liquid flow rate is controlled by a HPLC pump while the back-pressure regulator (BPR) maintains a constant pressure of CO_2 (8.5 atm) throughout the system.

Figure S2. ^{1}**H-NMR (500 MHz, CDCl$_3$),** ^{13}**C{^{1}H}-NMR (126 MHz, CDCl$_3$) of (2).**

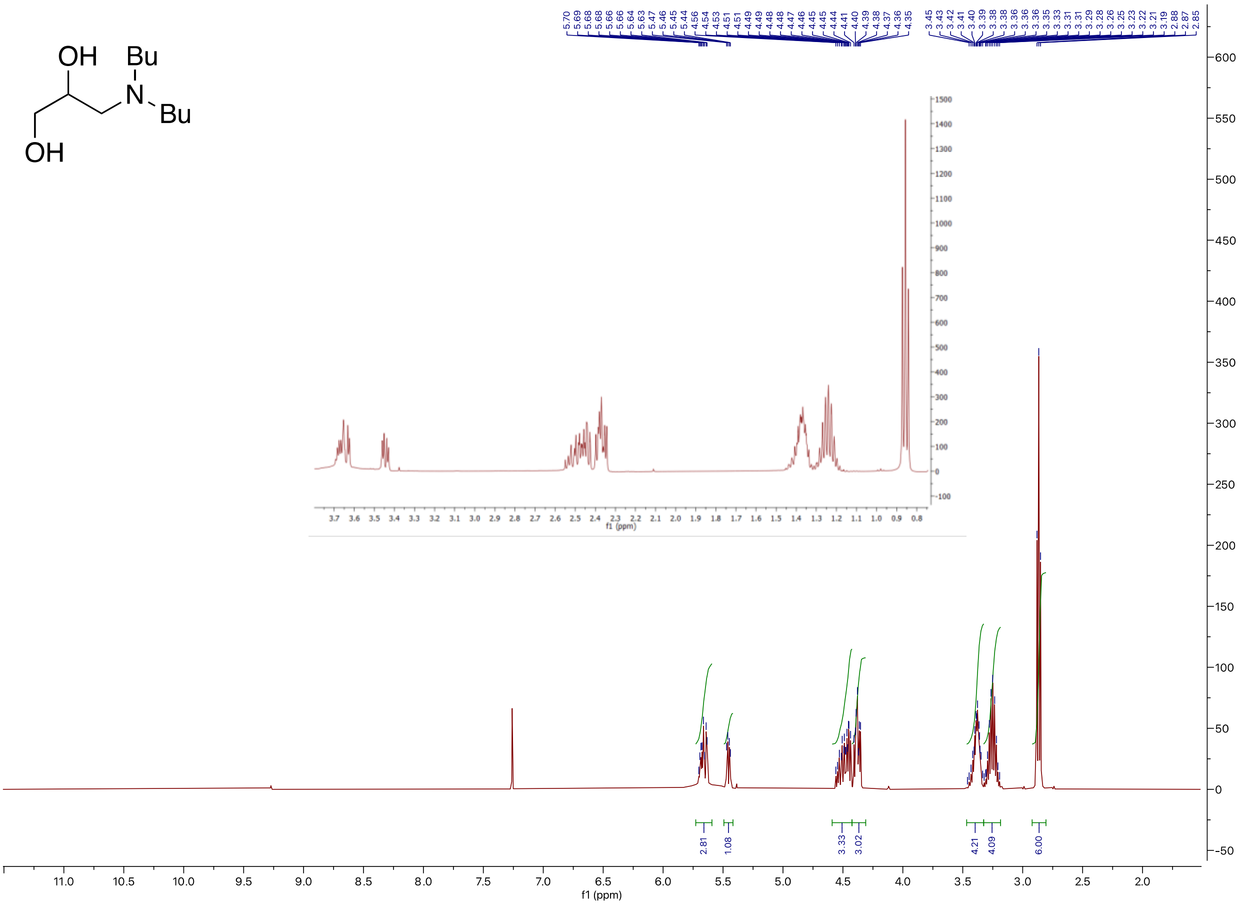

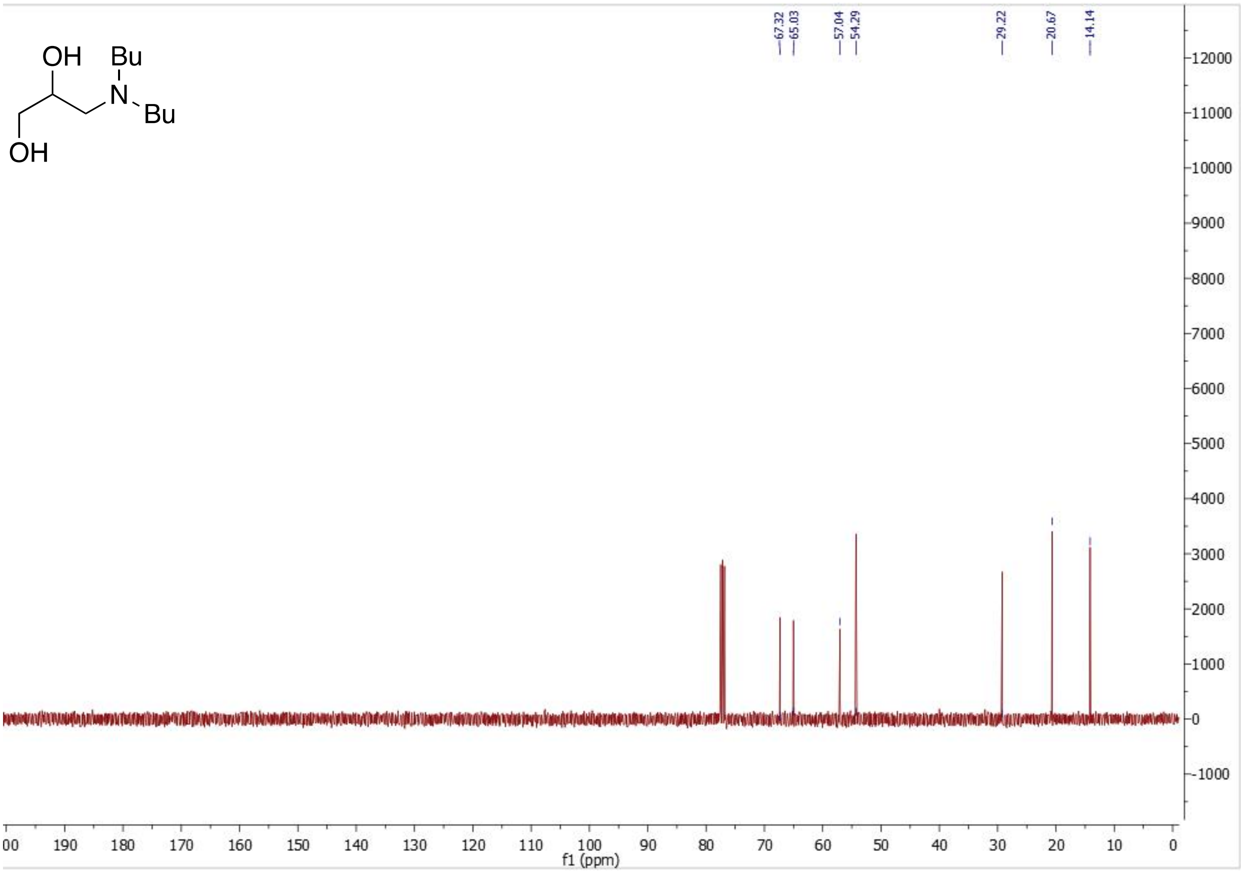

Figure S3. ^{1}H-NMR (300 MHz, DMSO-d$_6$), ^{13}C{^{1}H}-NMR (101 MHz, DMSO-d$_6$) of (3)

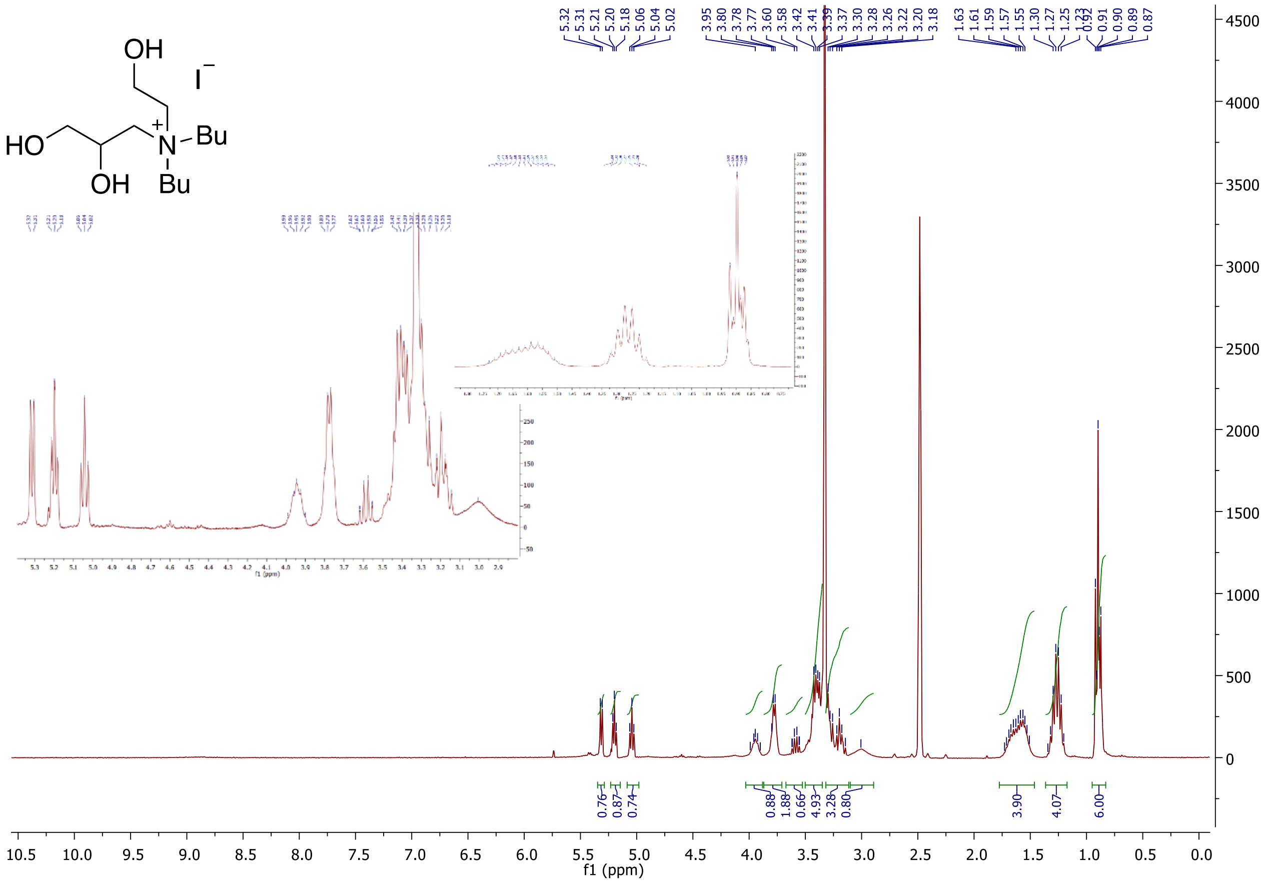

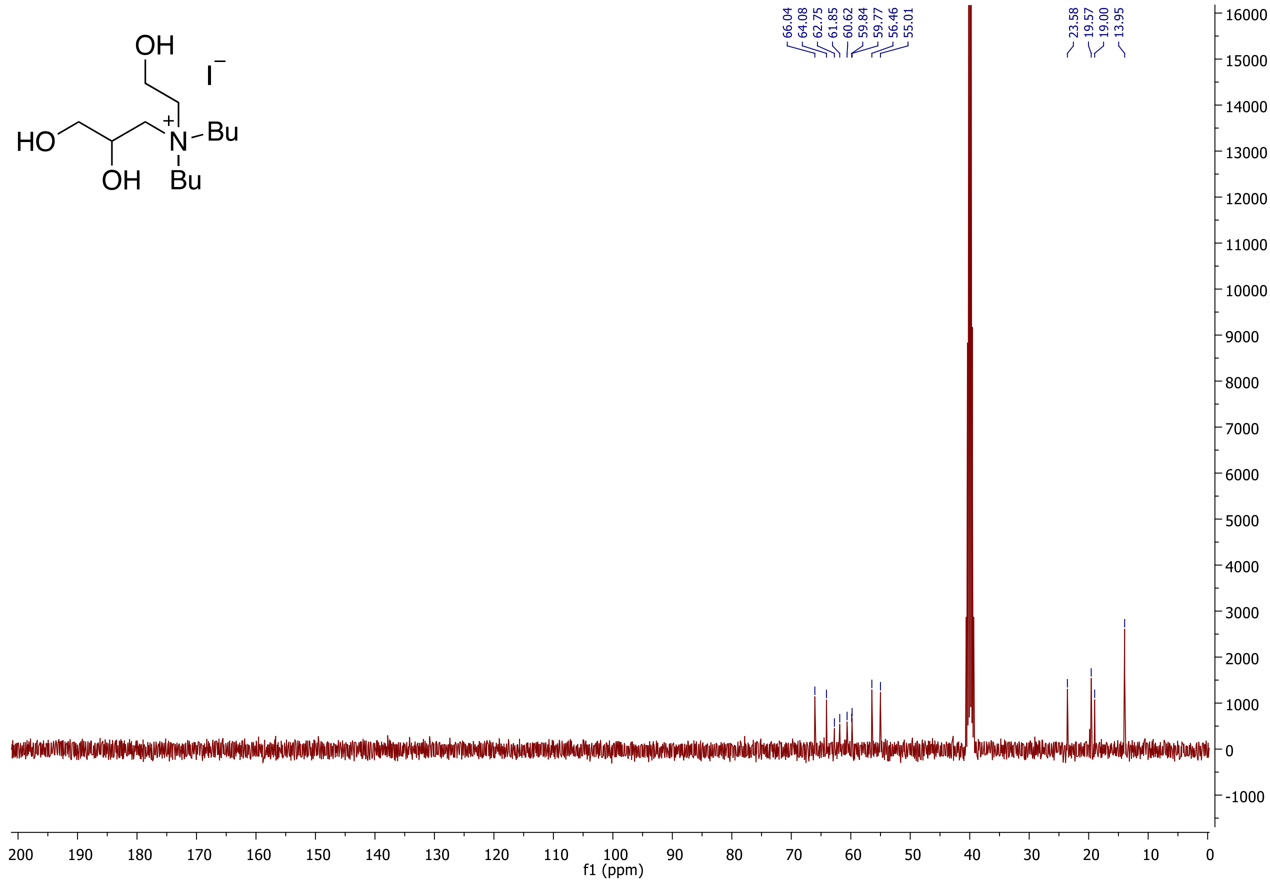

Figure S4. [1]**H-NMR (300 MHz, DMSO-d$_6$),** [13]**C{**[1]**H}-NMR (101 MHz, DMSO-d$_6$) of (5)**

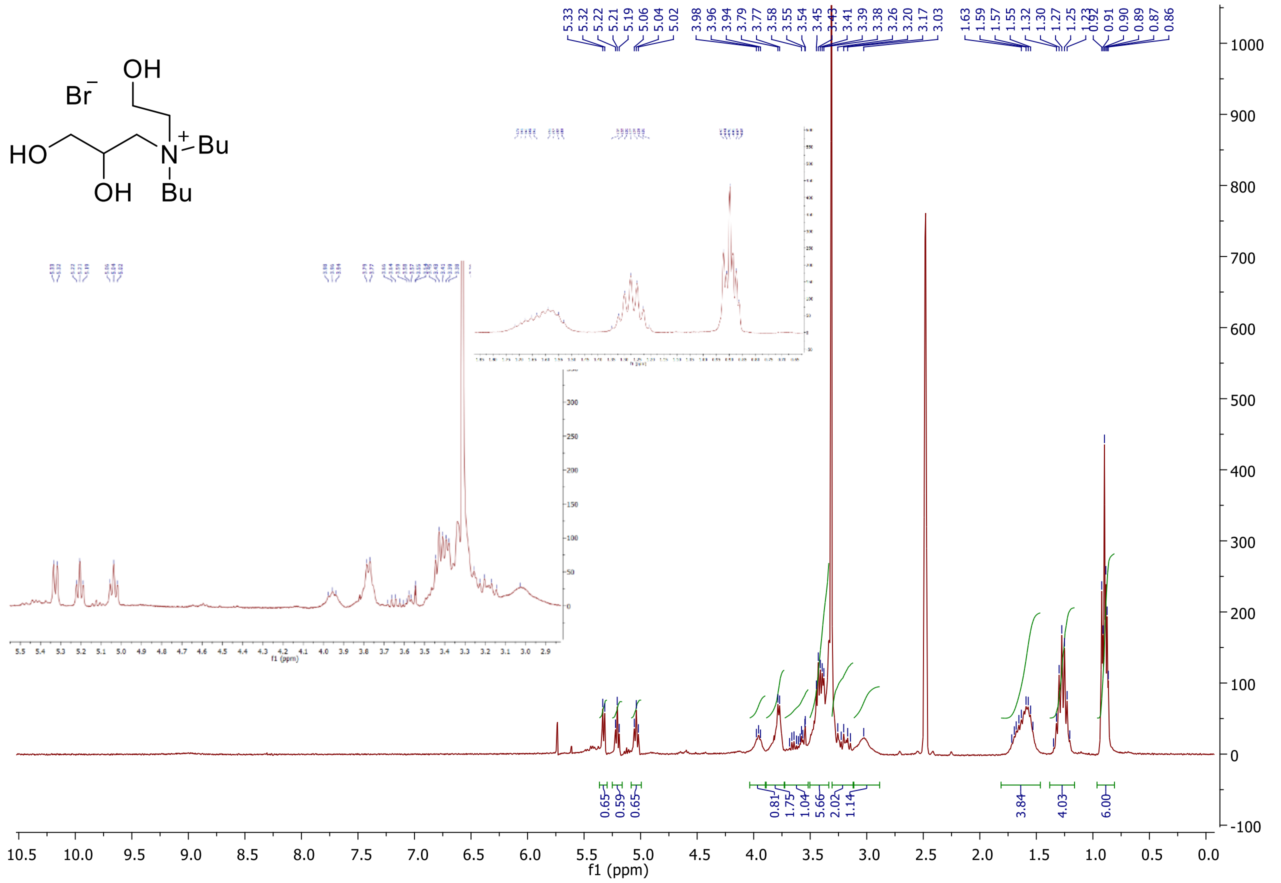

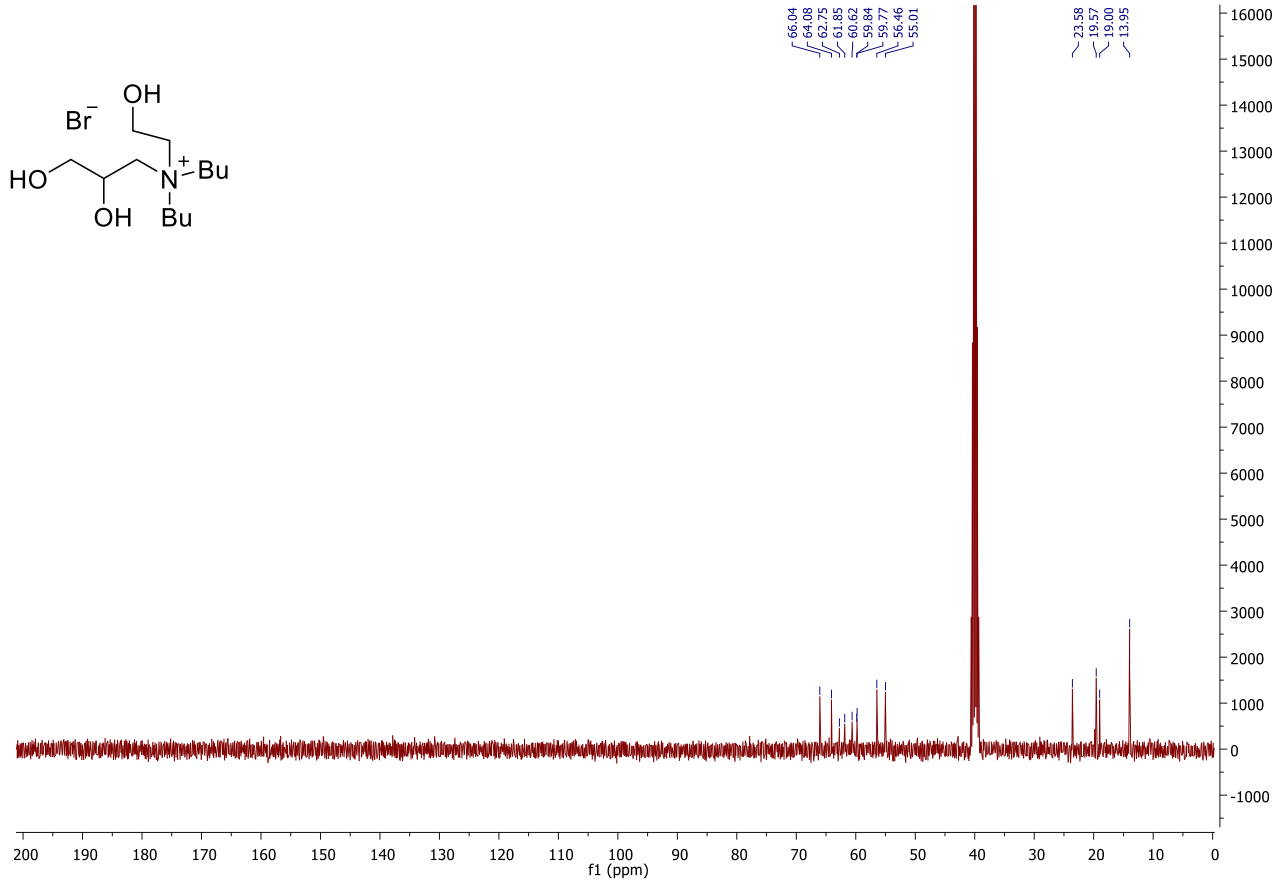

Figure S5. ^{1}H-NMR (500 MHz, D_2O), $^{13}C\{^{1}H\}$-NMR (126 MHz, D_2O) of (6)

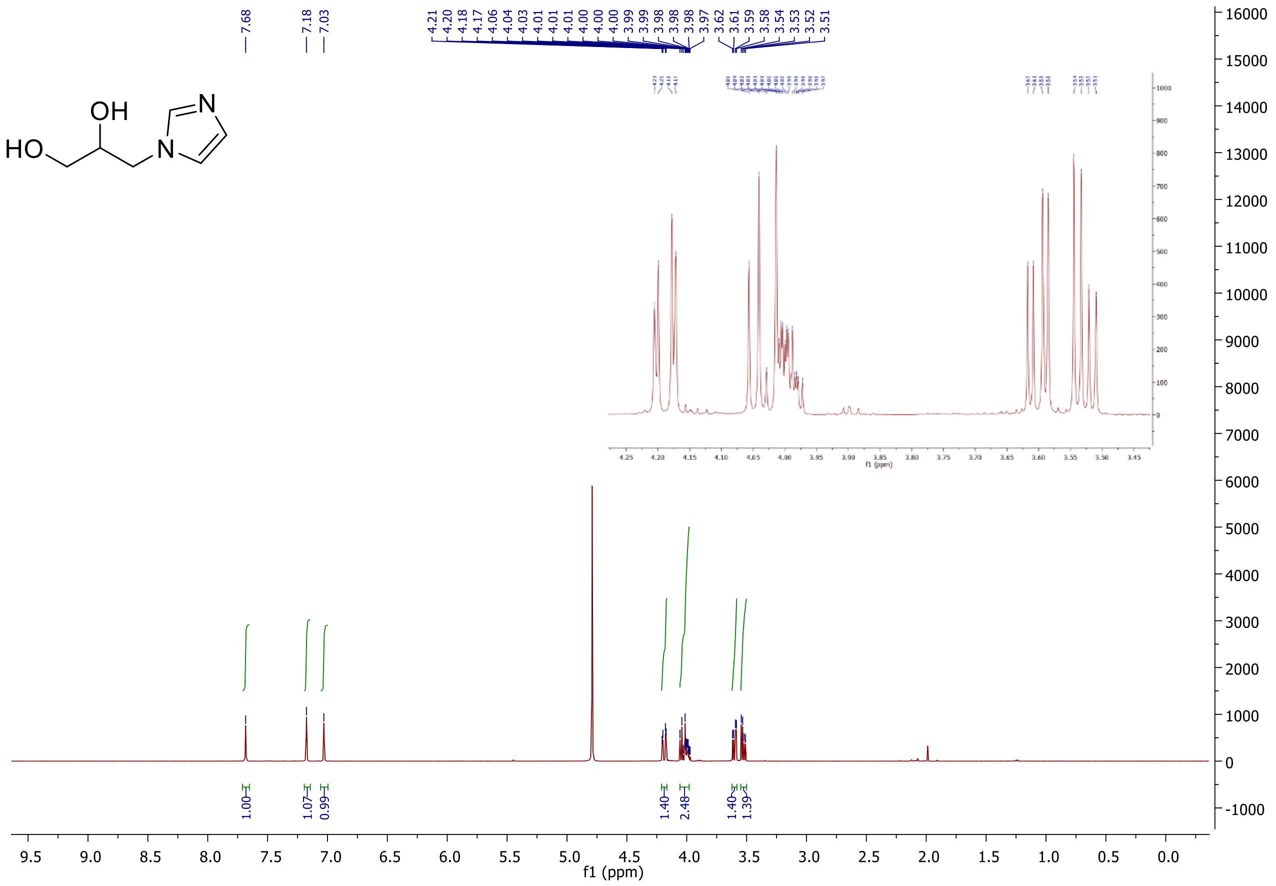

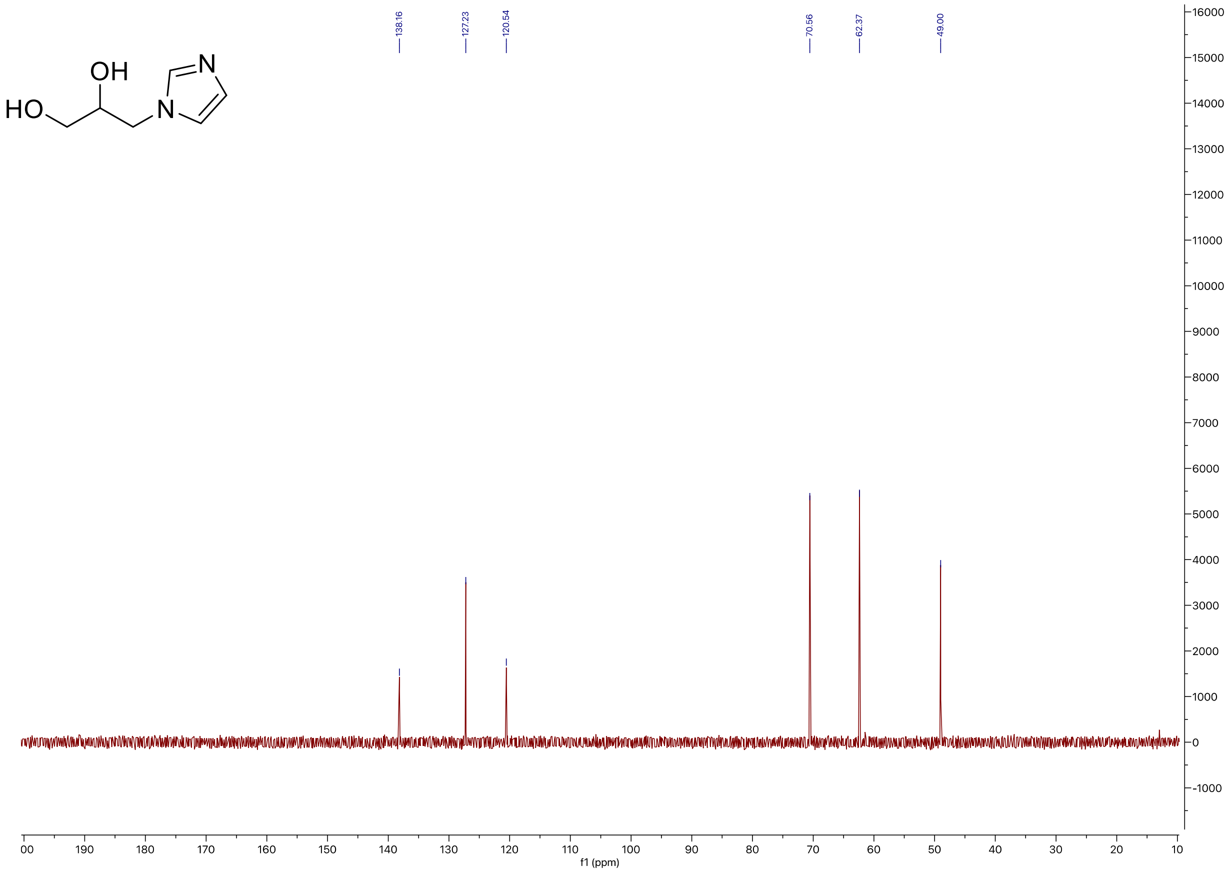

Figure S6. ^{1}H-NMR (300 MHz, DMSO-d$_6$), ^{13}C{^{1}H}-NMR (101 MHz, DMSO-d$_6$) of (**8**).

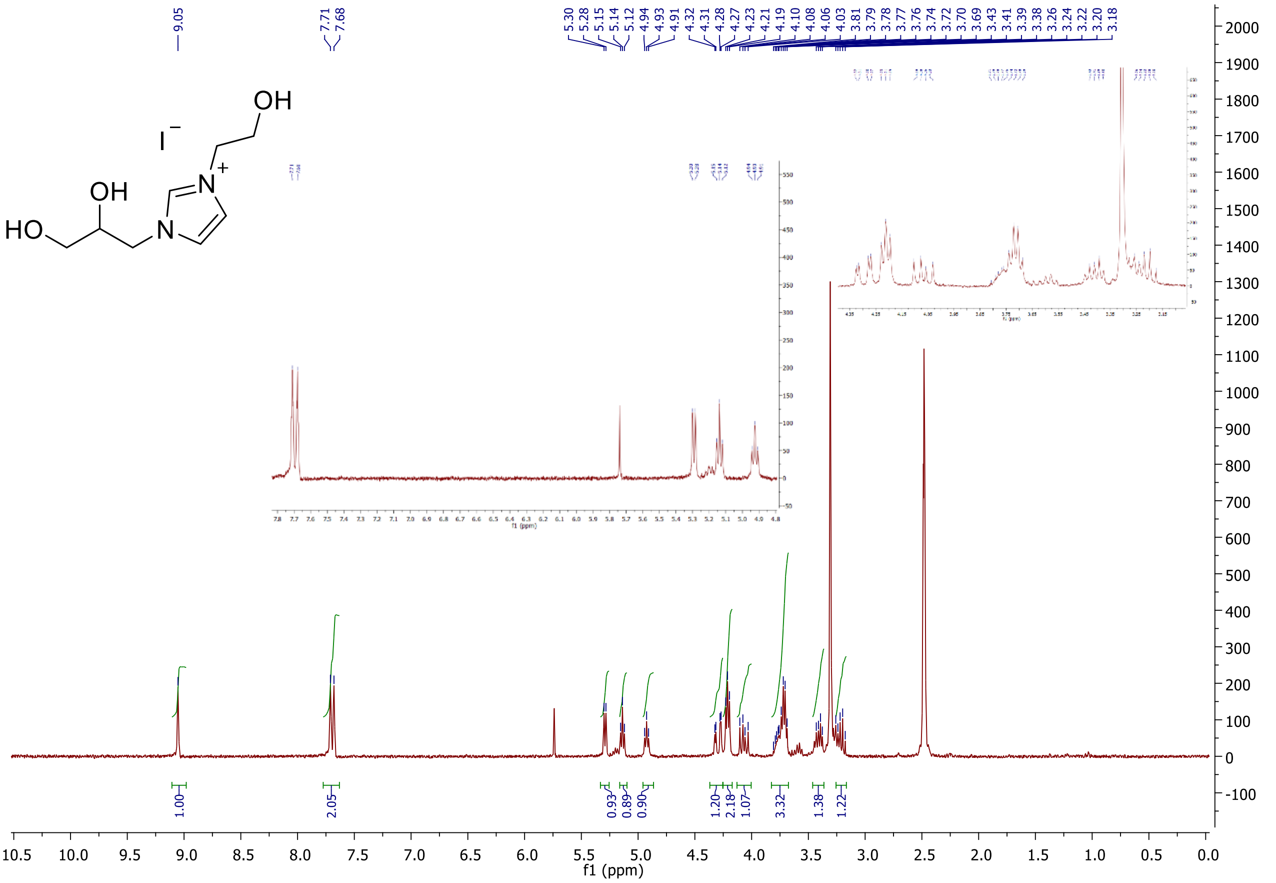

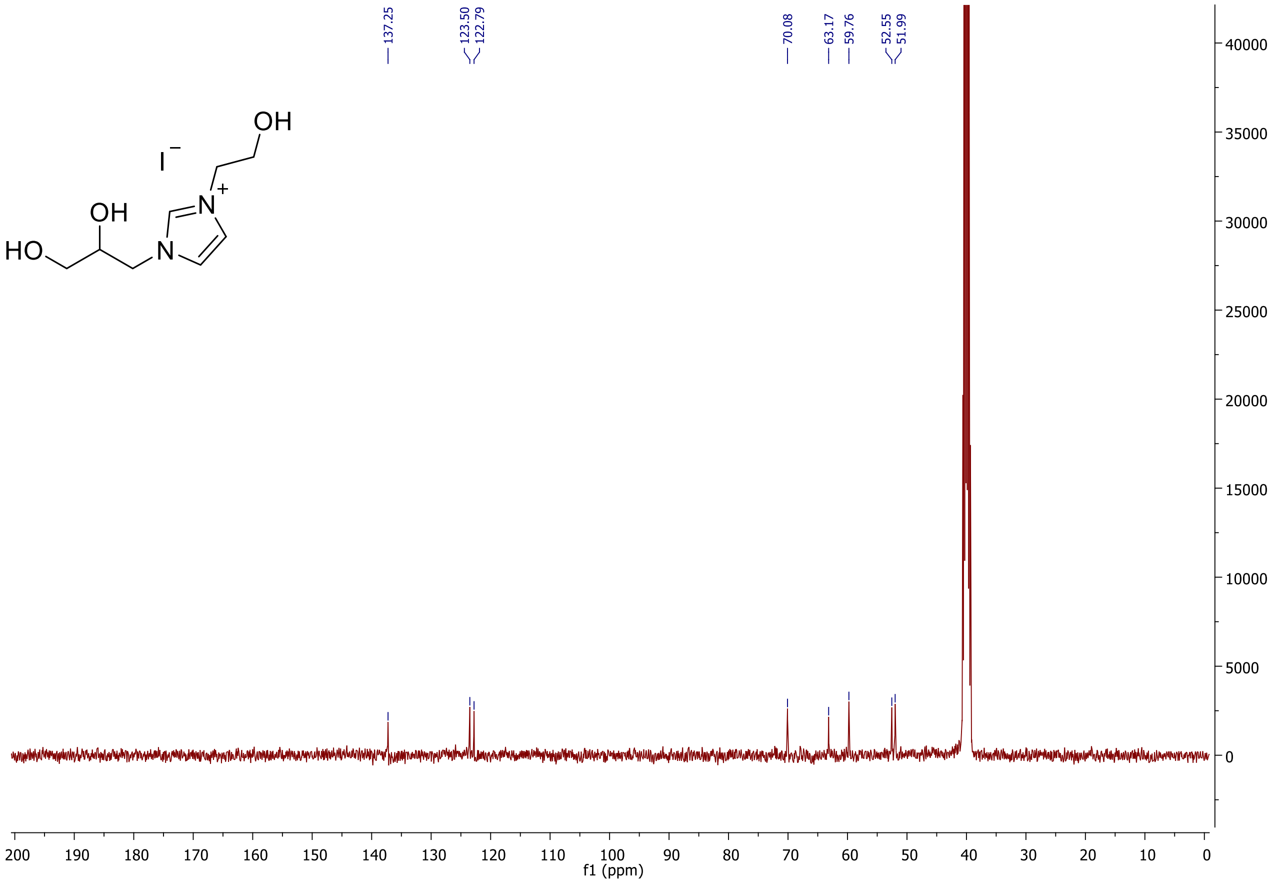

Figure S7. ^{1}**H-NMR (400 MHz, D**$_2$**O),** ^{13}**C{**^{1}**H}-NMR (101 MHz, D**$_2$**O) of (9)**.

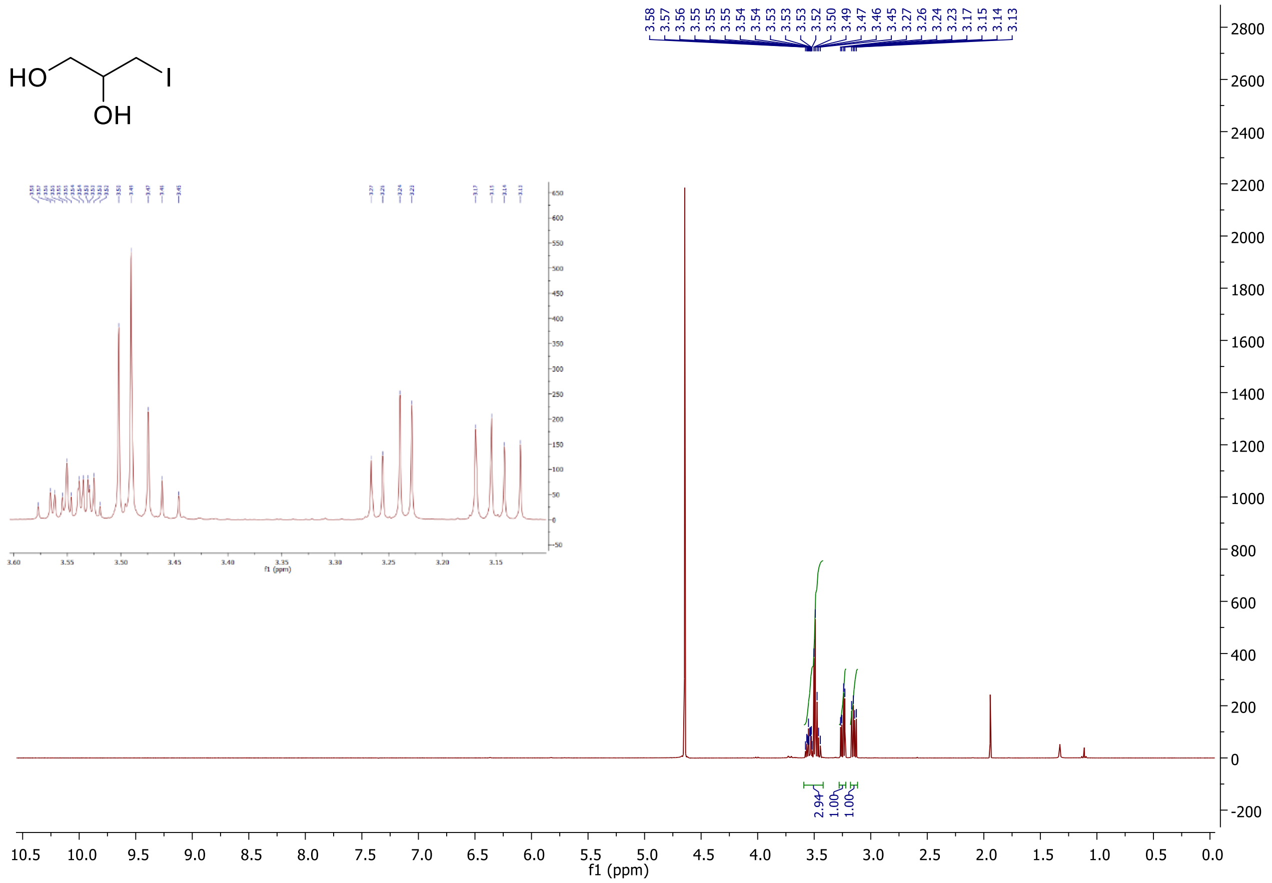

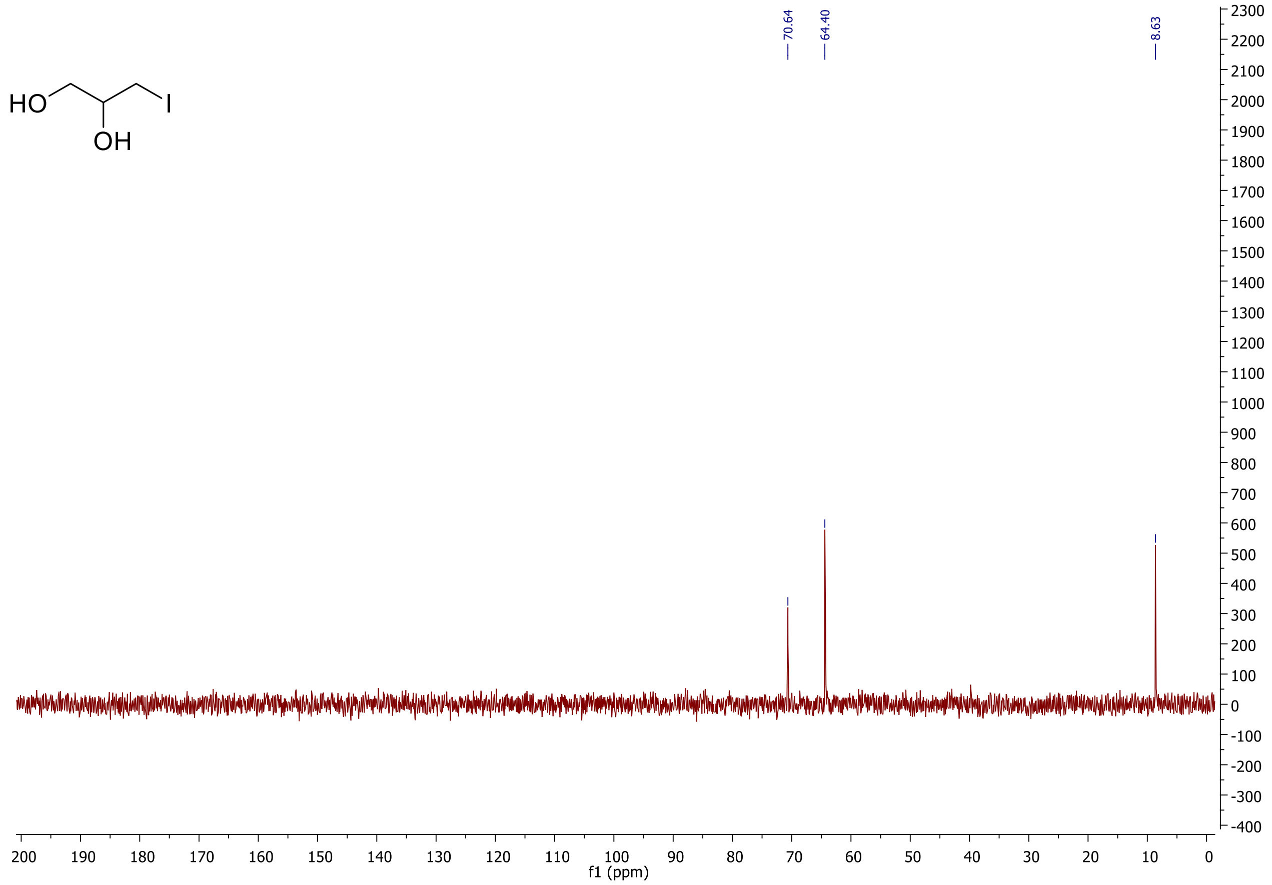

Figure S8. ^{1}H-NMR (400 MHz, $CDCl_3$), $^{13}C\{^{1}H\}$-NMR (101 MHz, $CDCl_3$) of (13).

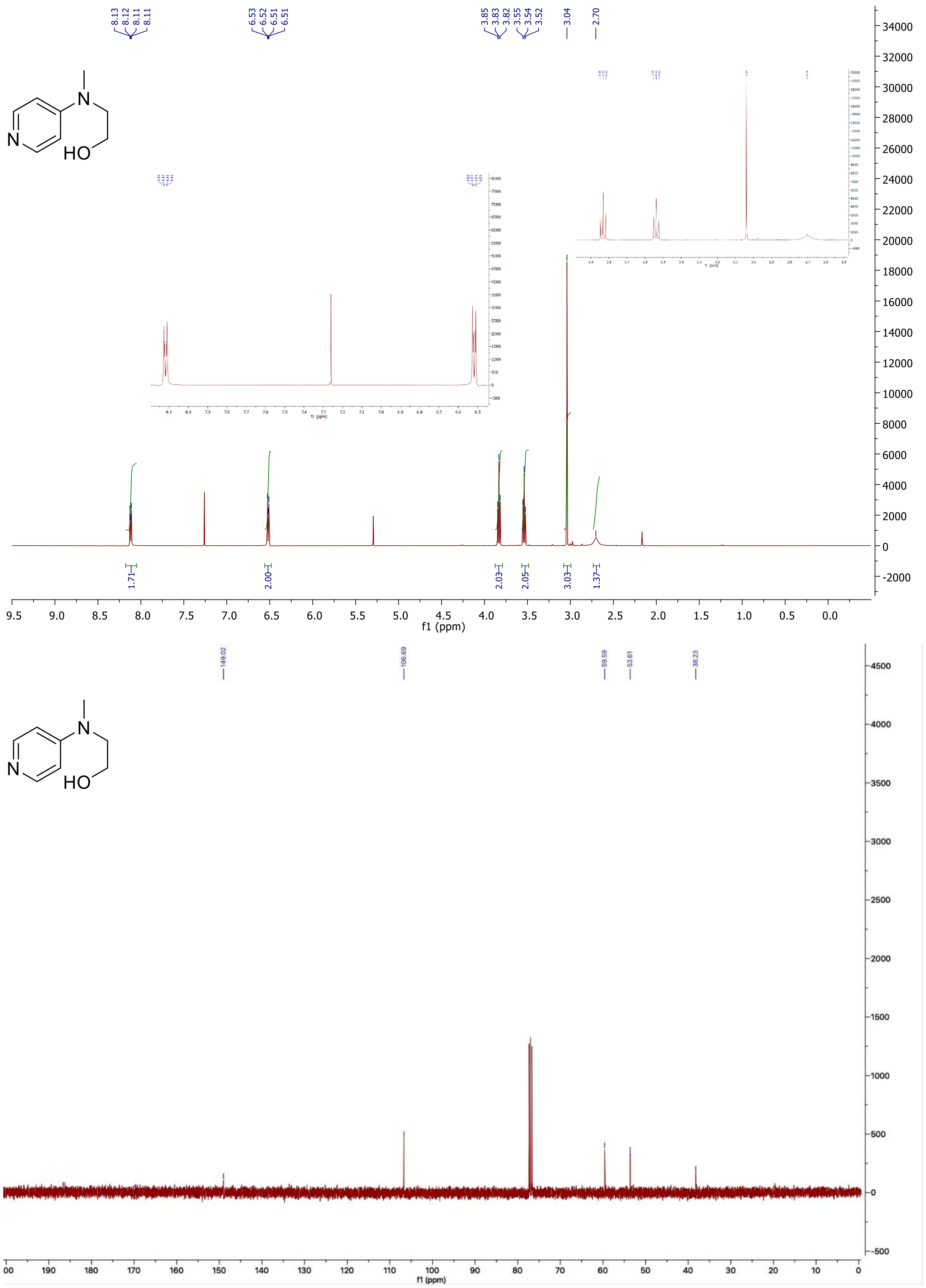

Figure S9. ^{1}H-NMR (300 MHz, DMSO-d$_6$), ^{13}C{^{1}H}-NMR (101 MHz, DMSO-d$_6$) of (14)

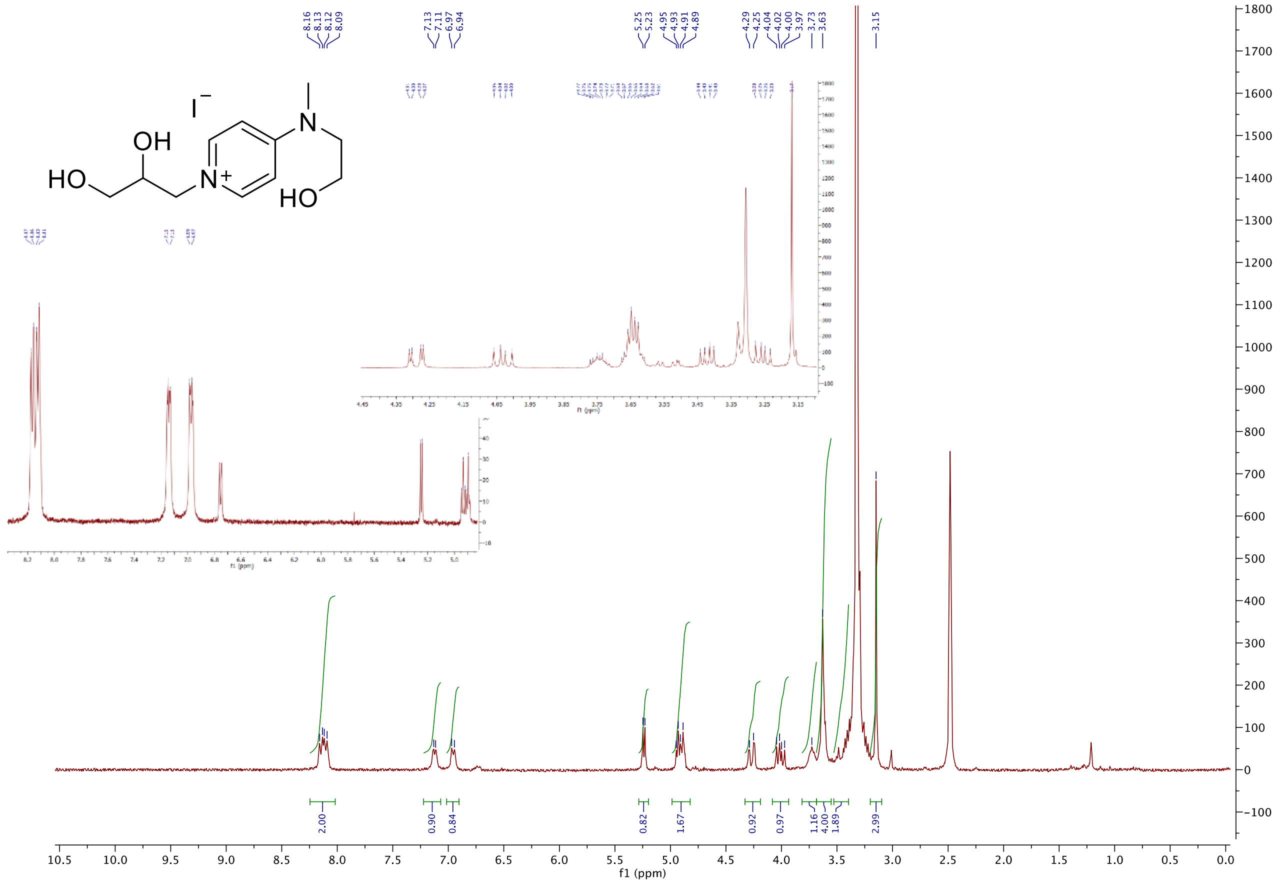

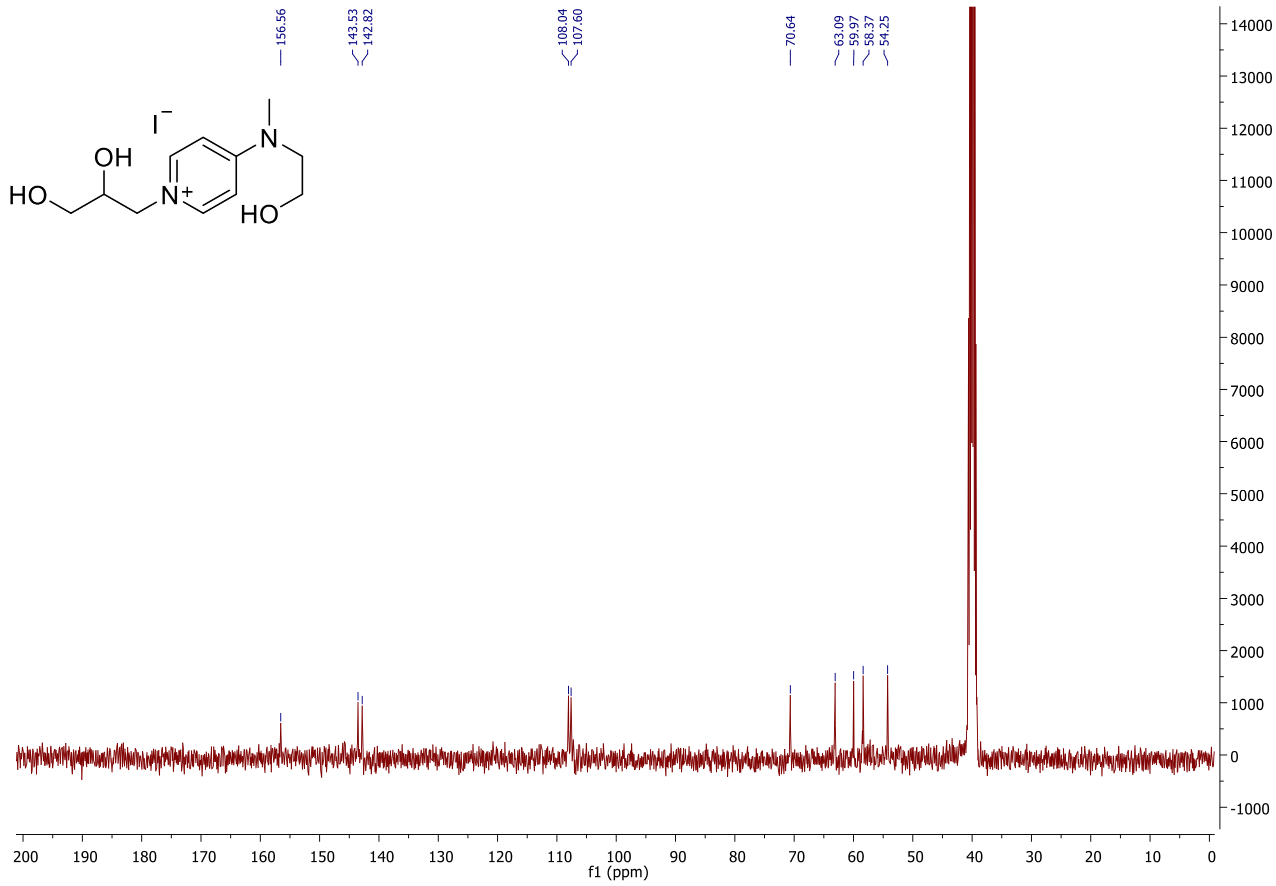

Figure S10. ^{1}H-NMR (300 MHz, $CDCl_3$), ^{13}C{^{1}H}-NMR (101 MHz, $CDCl_3$) of (**15**).

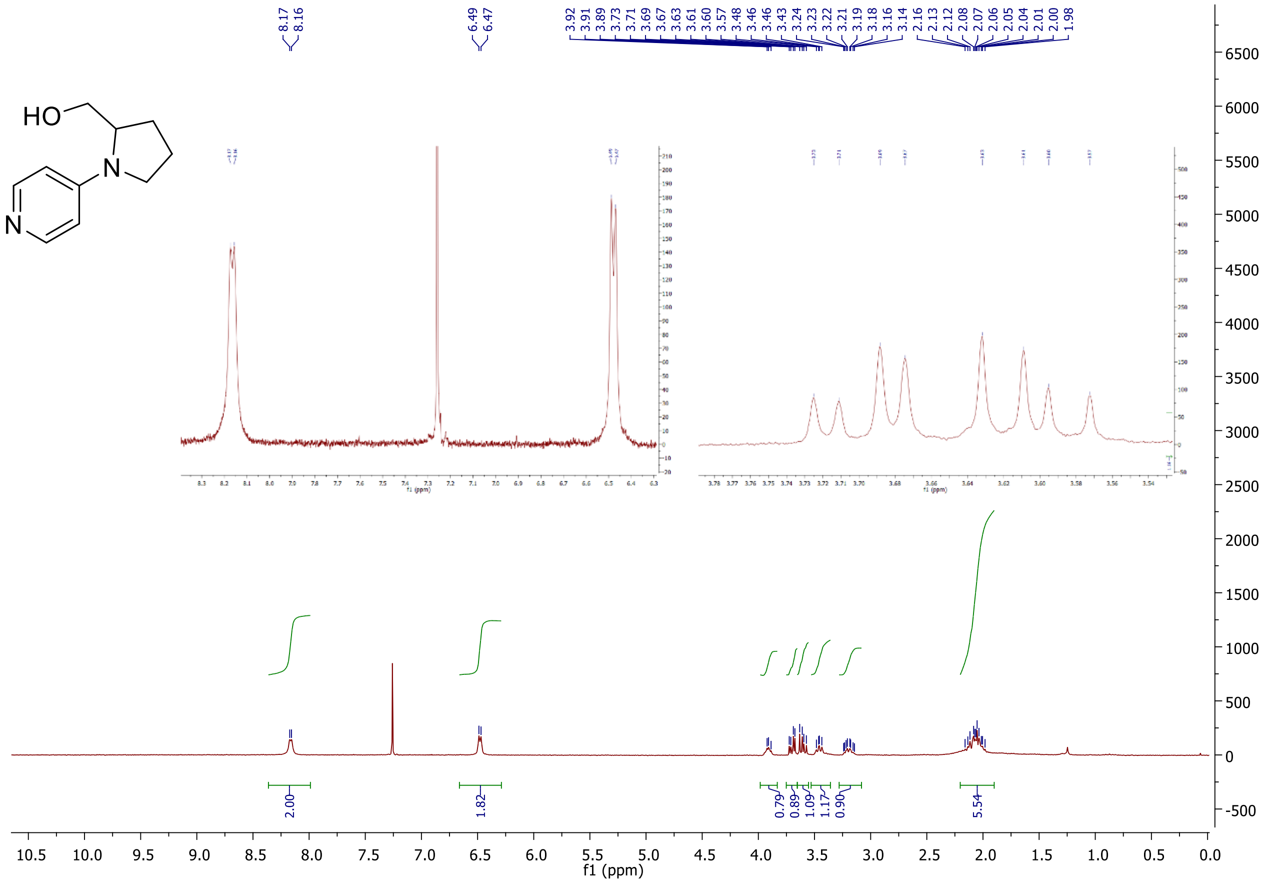

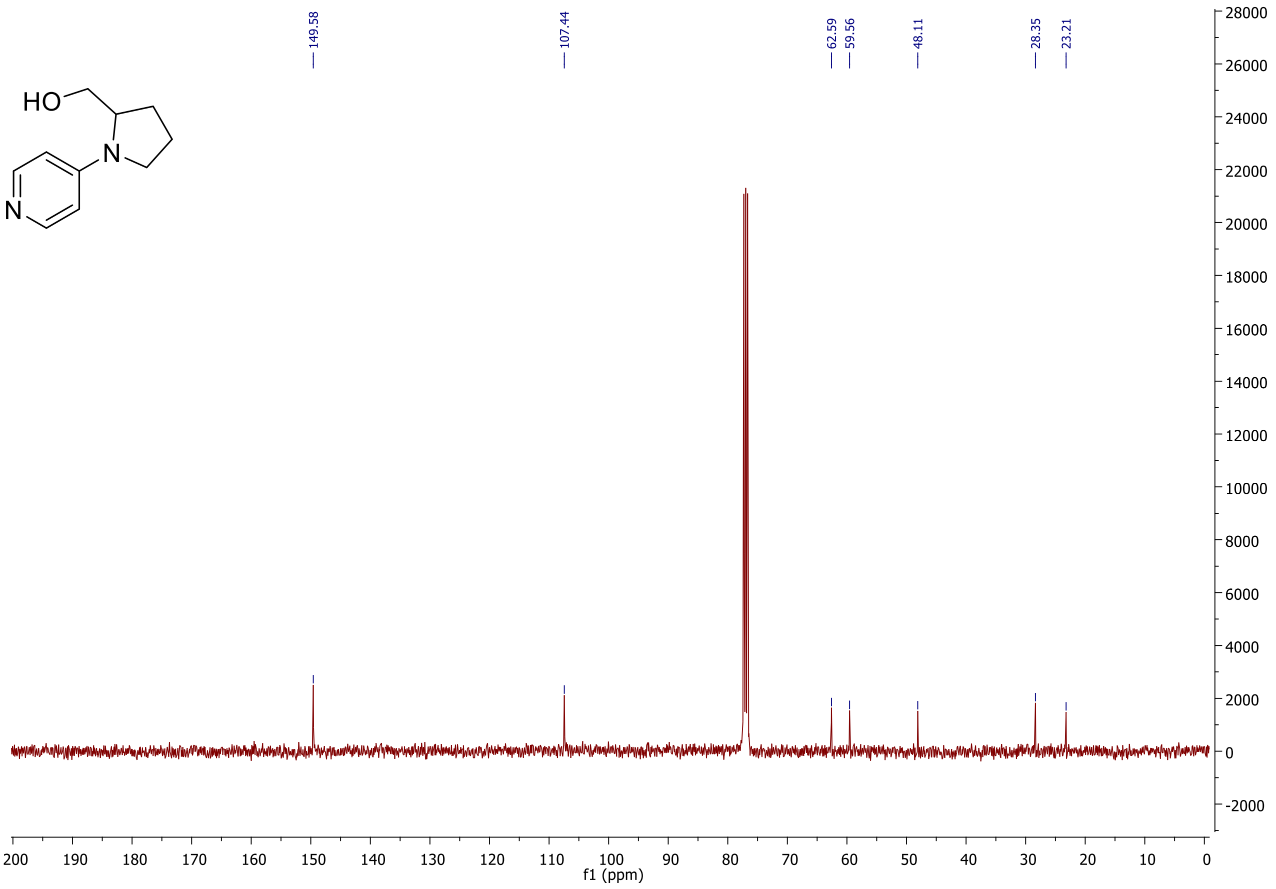

Figure S11. **^{1}H-NMR (300 MHz, DMSO-d$_6$), $^{13}C\{^{1}H\}$-NMR (101 MHz, DMSO-d$_6$) of (16)**.

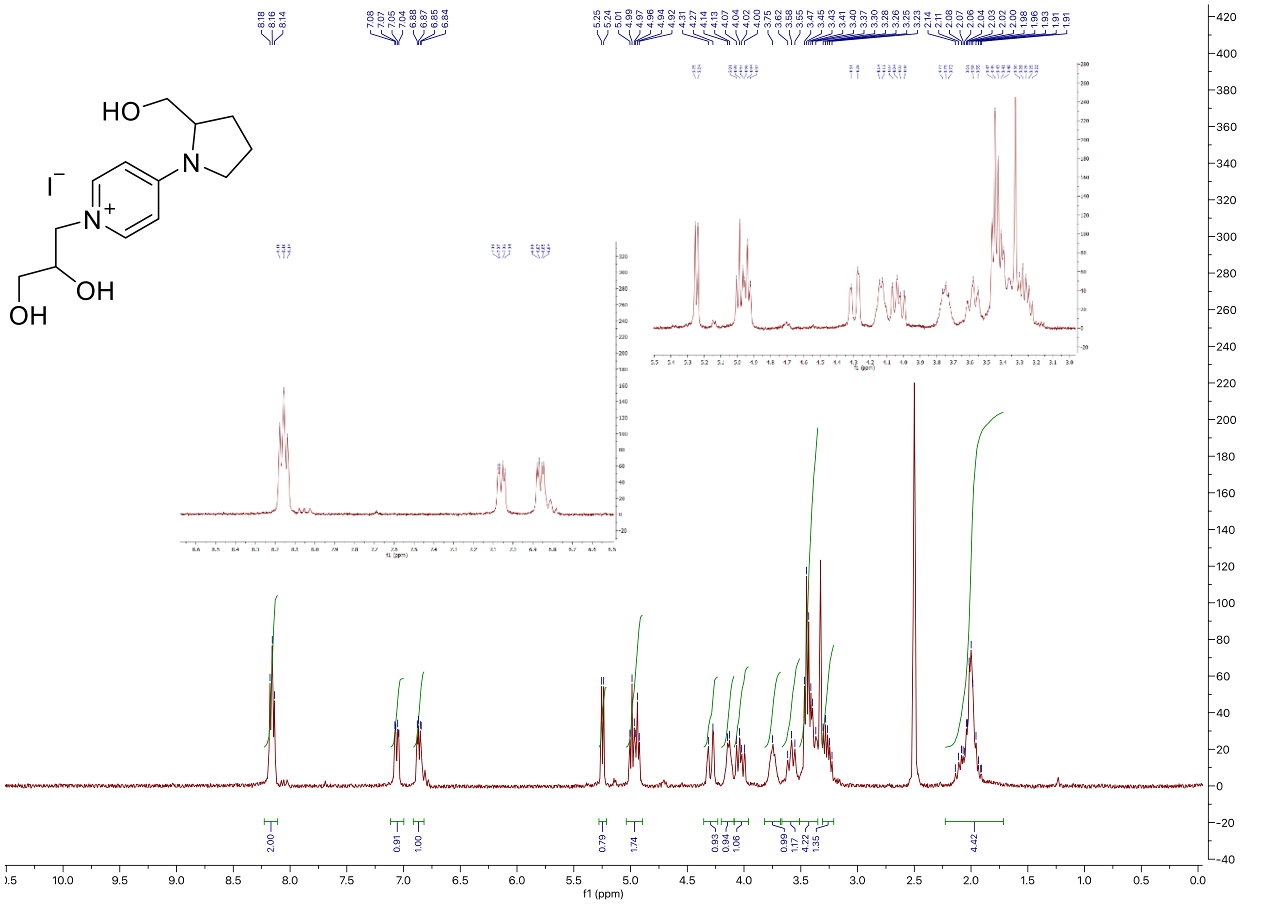

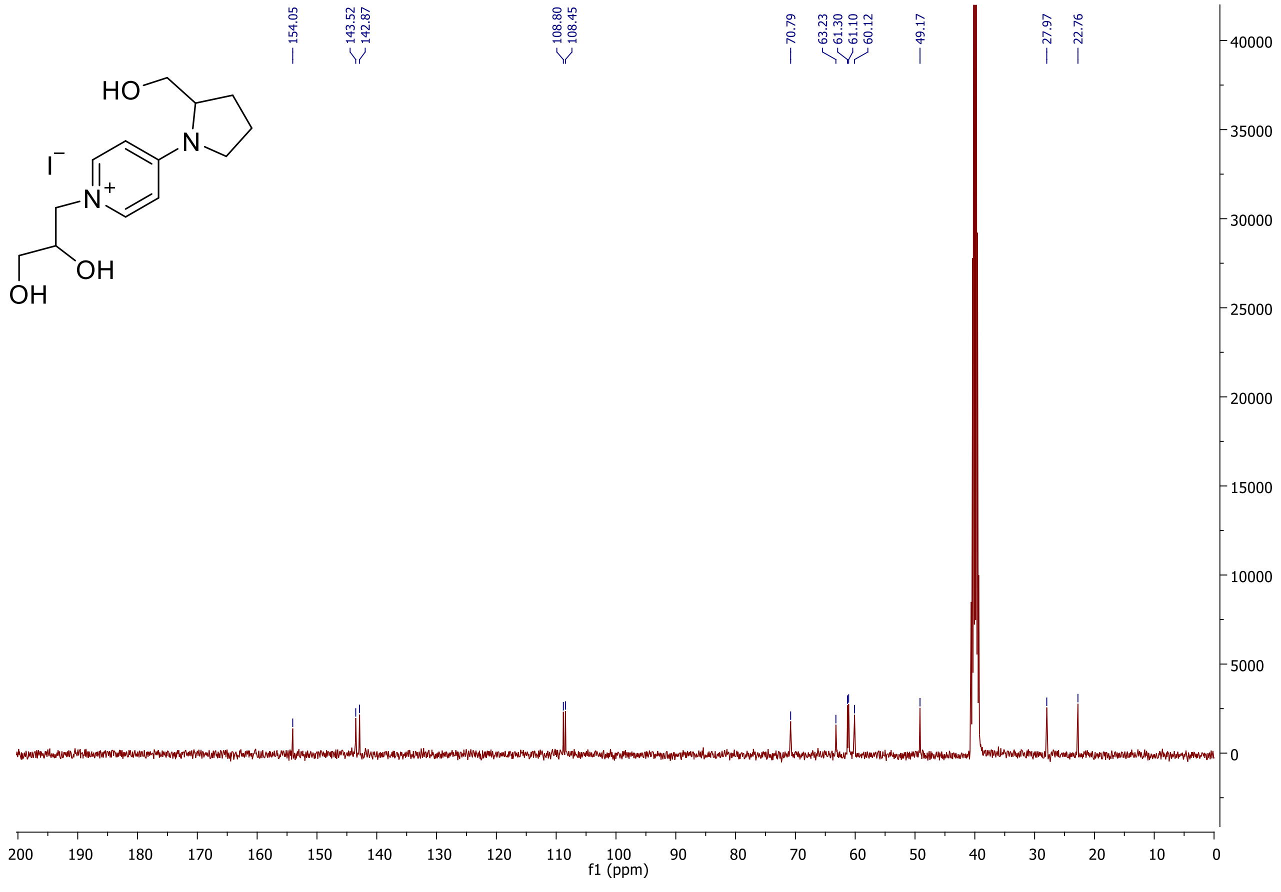

Figure S12. ^{1}H-NMR (500 MHz, $CDCl_3$), ^{13}C{^{1}H}-NMR (126 MHz, $CDCl_3$) of (**18a**).

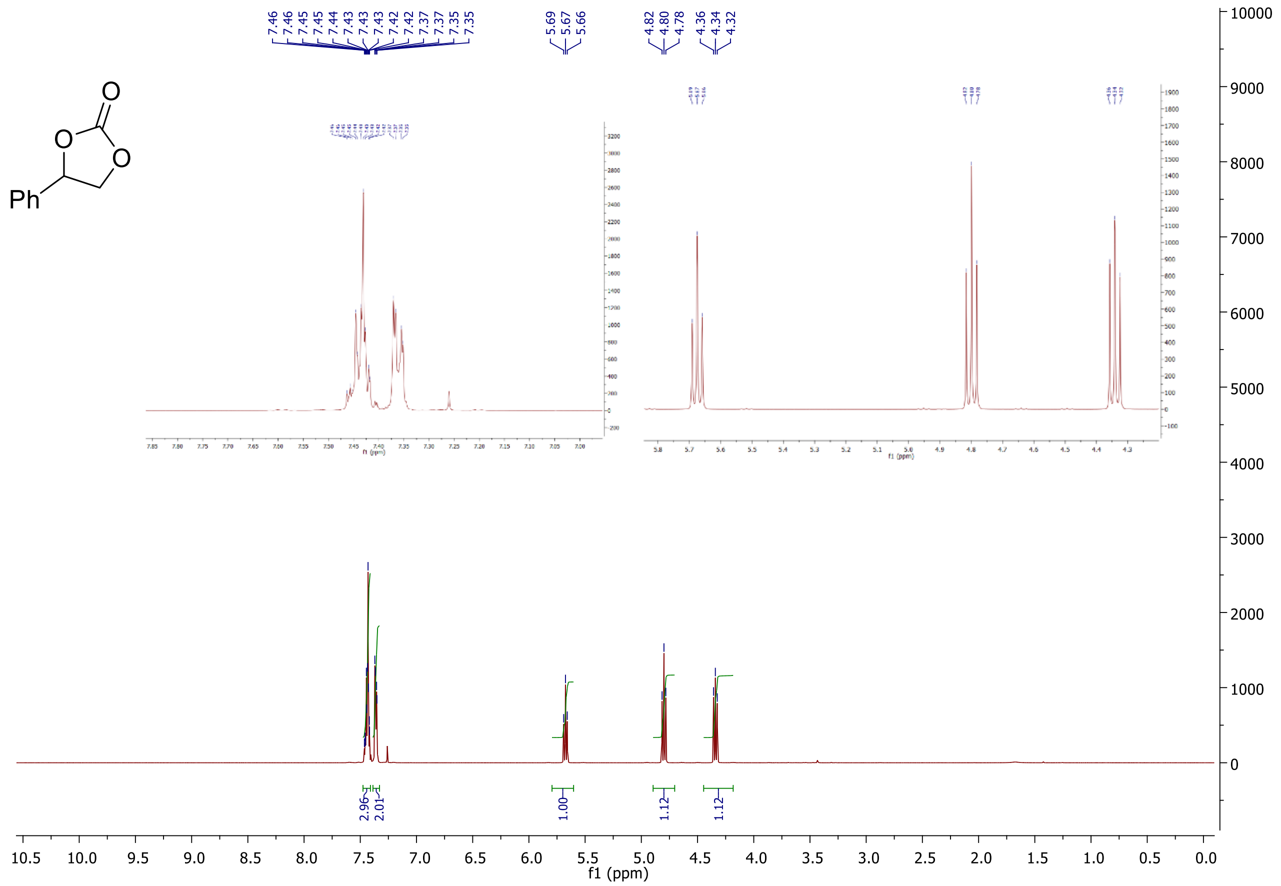

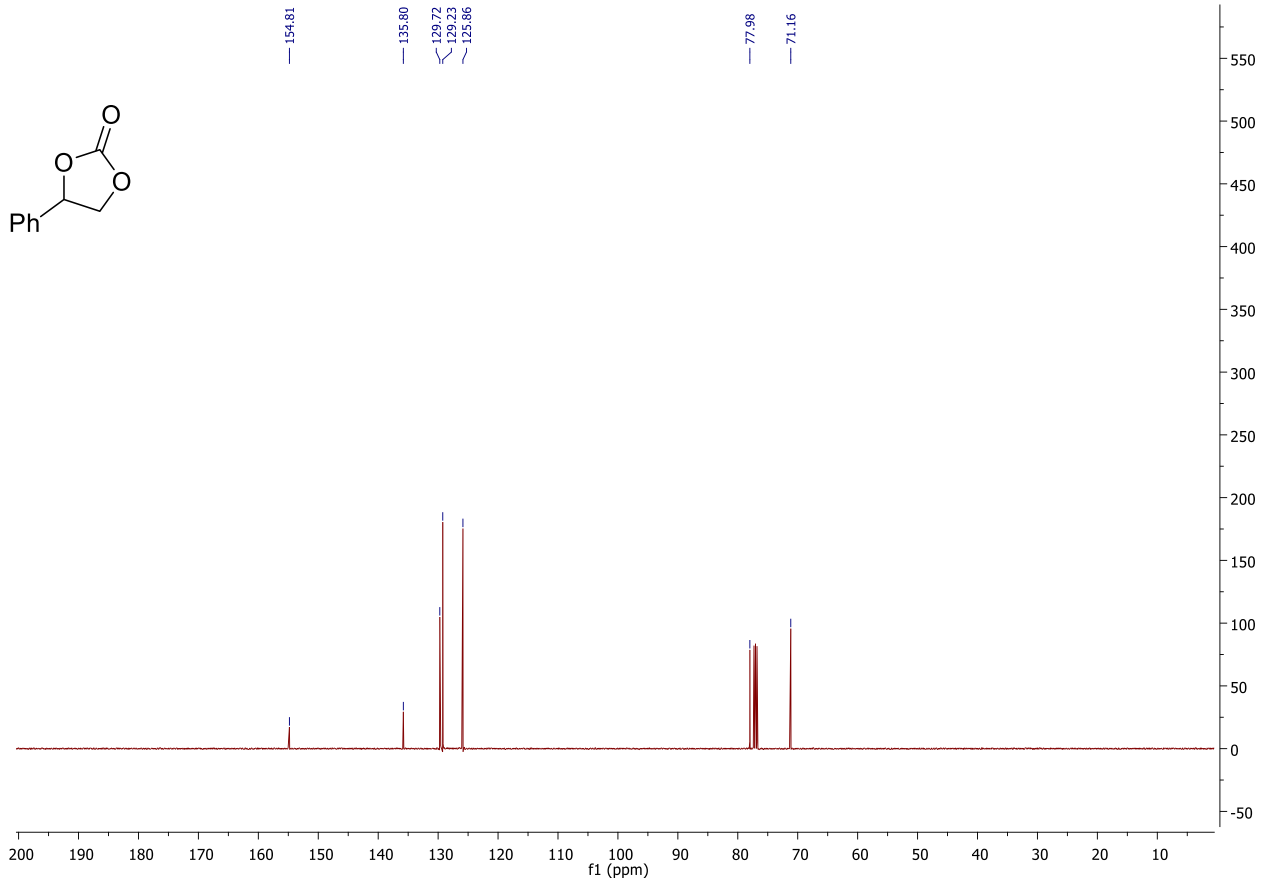

Figure S13. ^{1}H-NMR (300 MHz, $CDCl_3$), ^{13}C{^{1}H}-NMR (101 MHz, $CDCl_3$) of (18b).

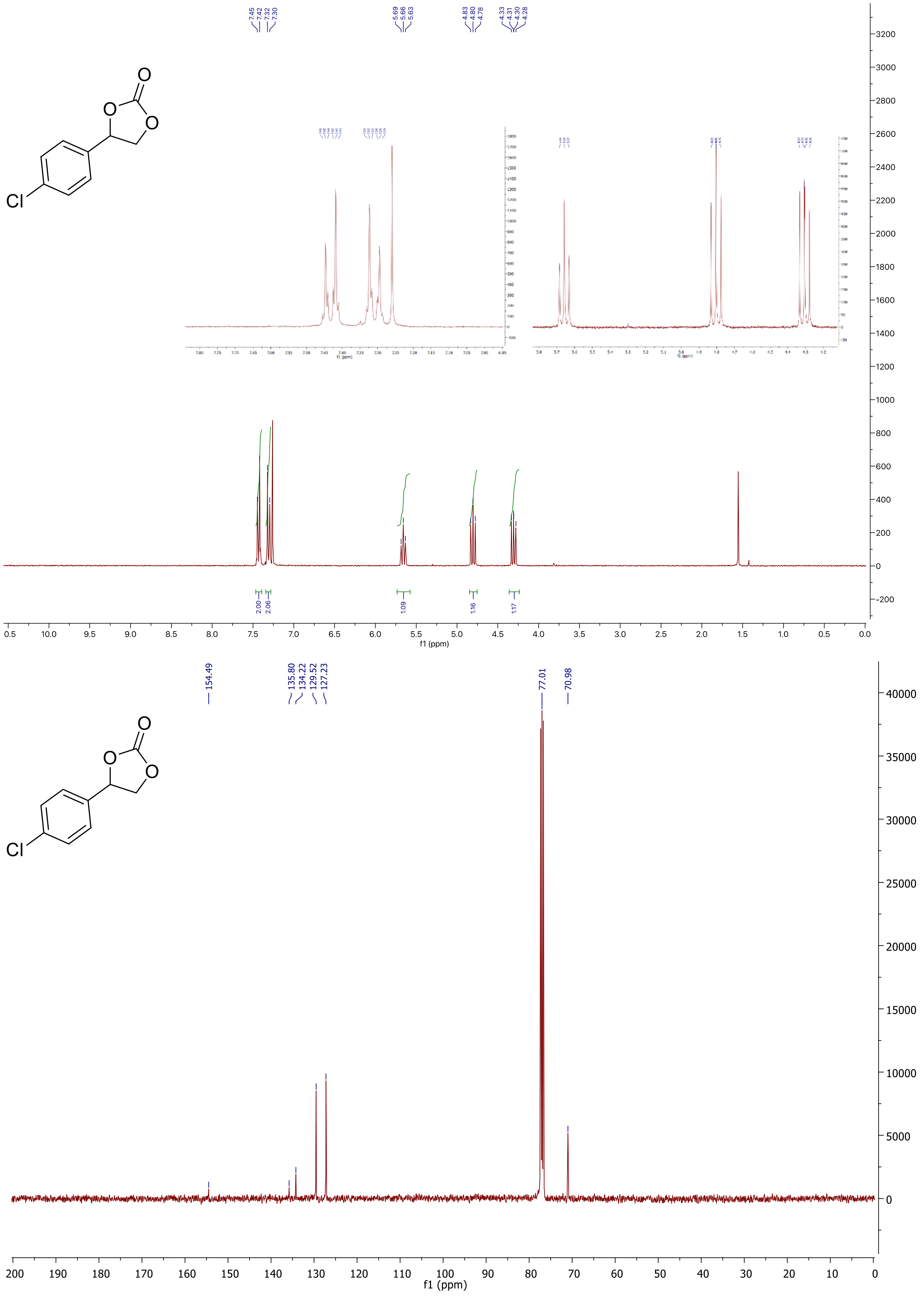

Figure S14. ^{1}H-NMR (400 MHz, $CDCl_3$), $^{13}C\{^1H\}$-NMR (101 MHz, $CDCl_3$) of (**18c**).

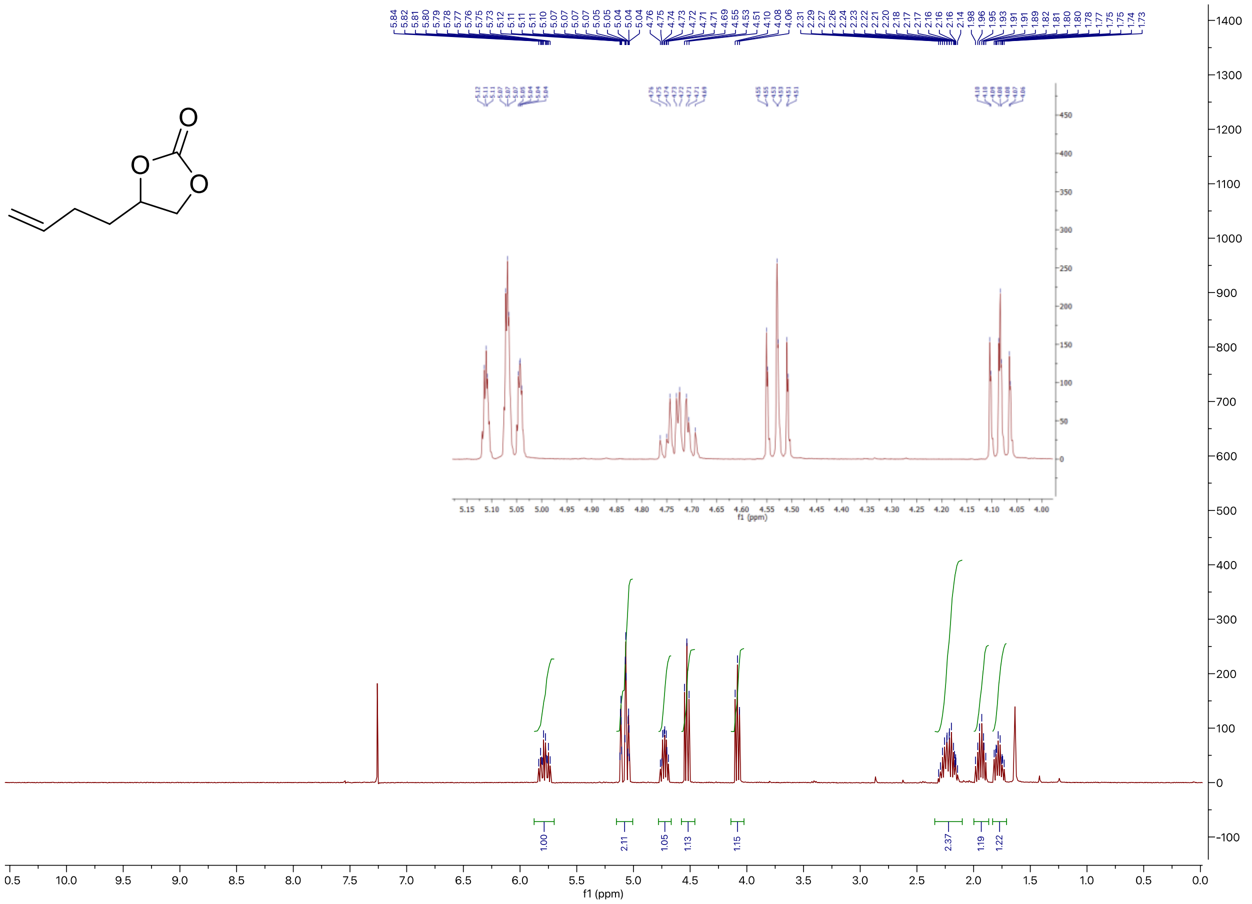

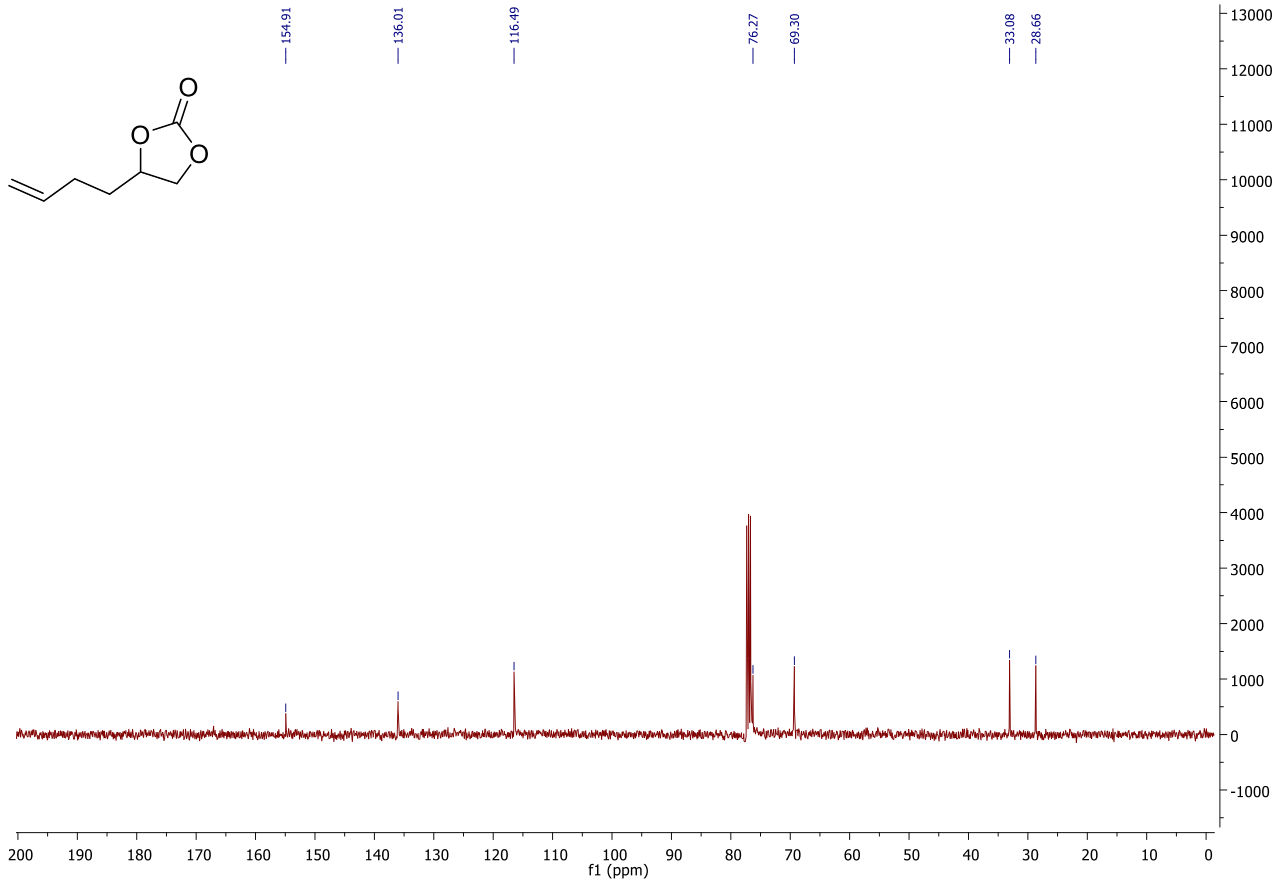

Figure S15. ^{1}**H-NMR (400 MHz, CDCl$_3$),** ^{13}**C{^{1}H}-NMR (101 MHz, CDCl$_3$) of (18d)**.

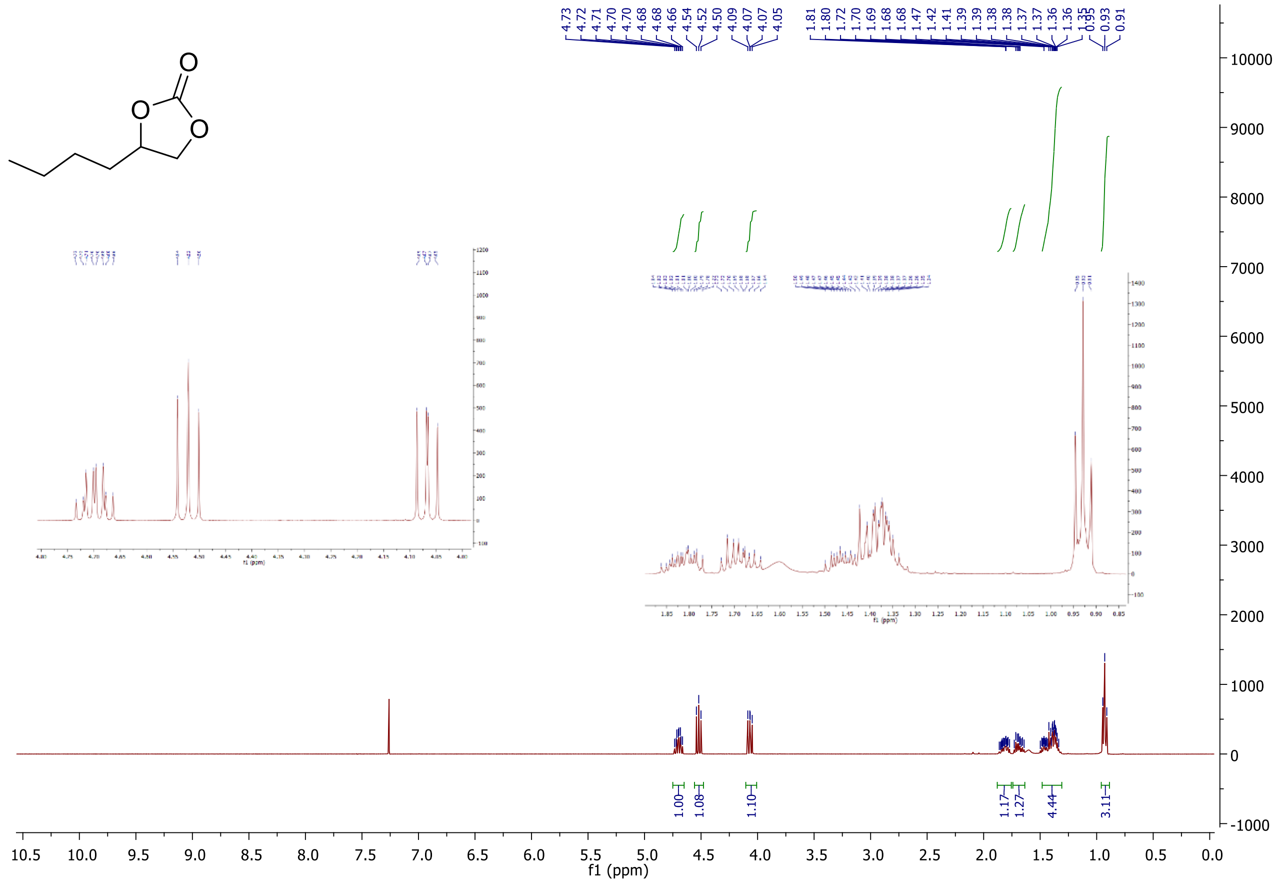

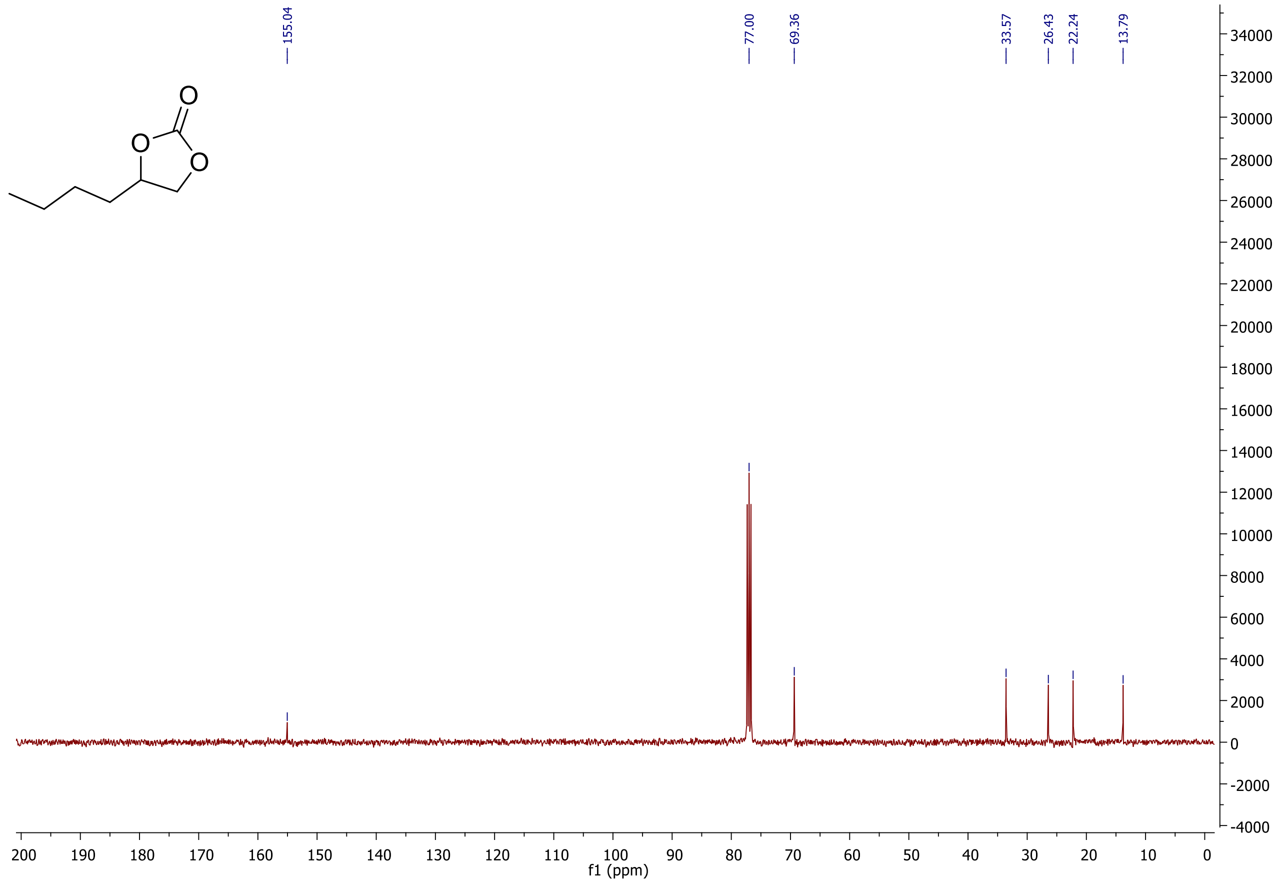

Figure S16. ^{1}H-NMR (400 MHz, $CDCl_3$), ^{13}C{^{1}H}-NMR (101 MHz, $CDCl_3$) of (**18e**).

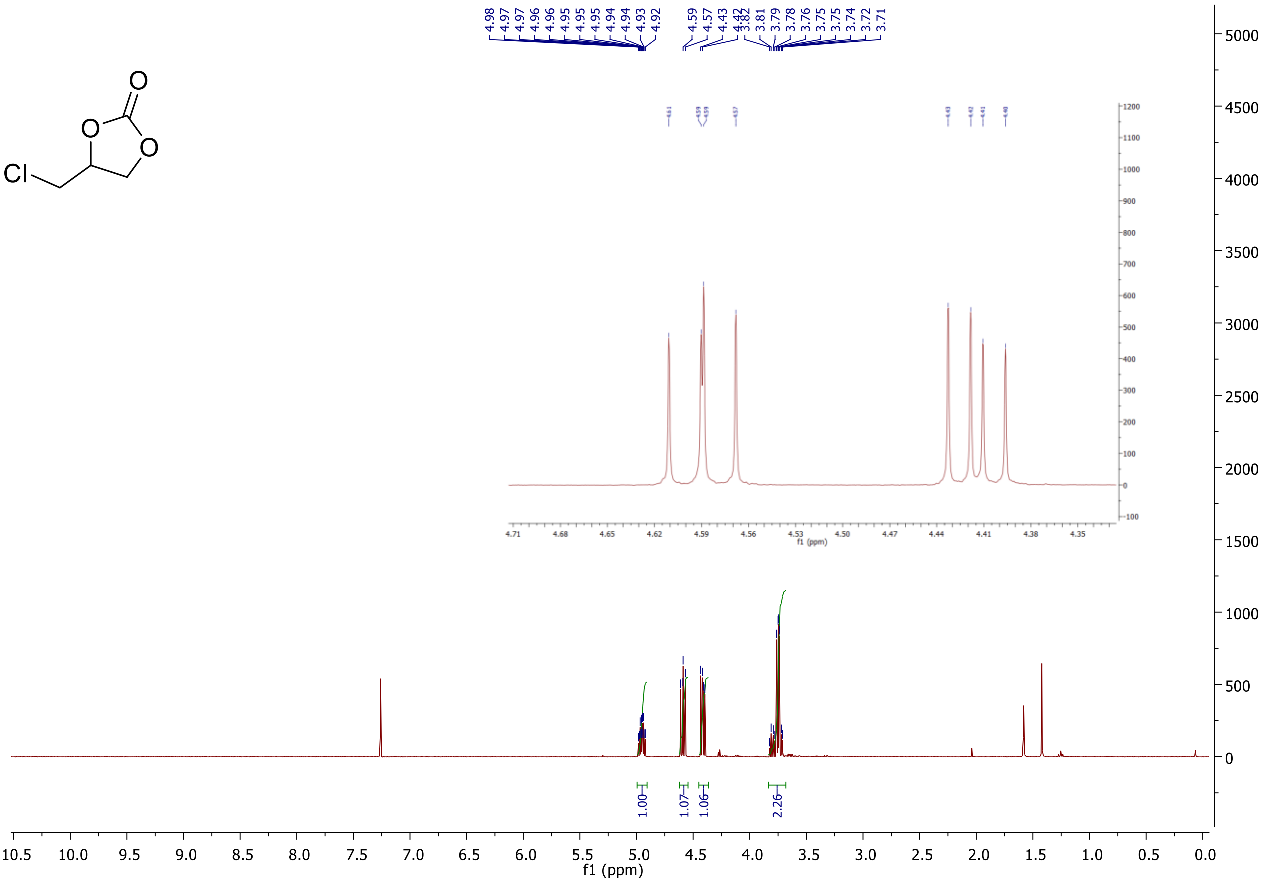

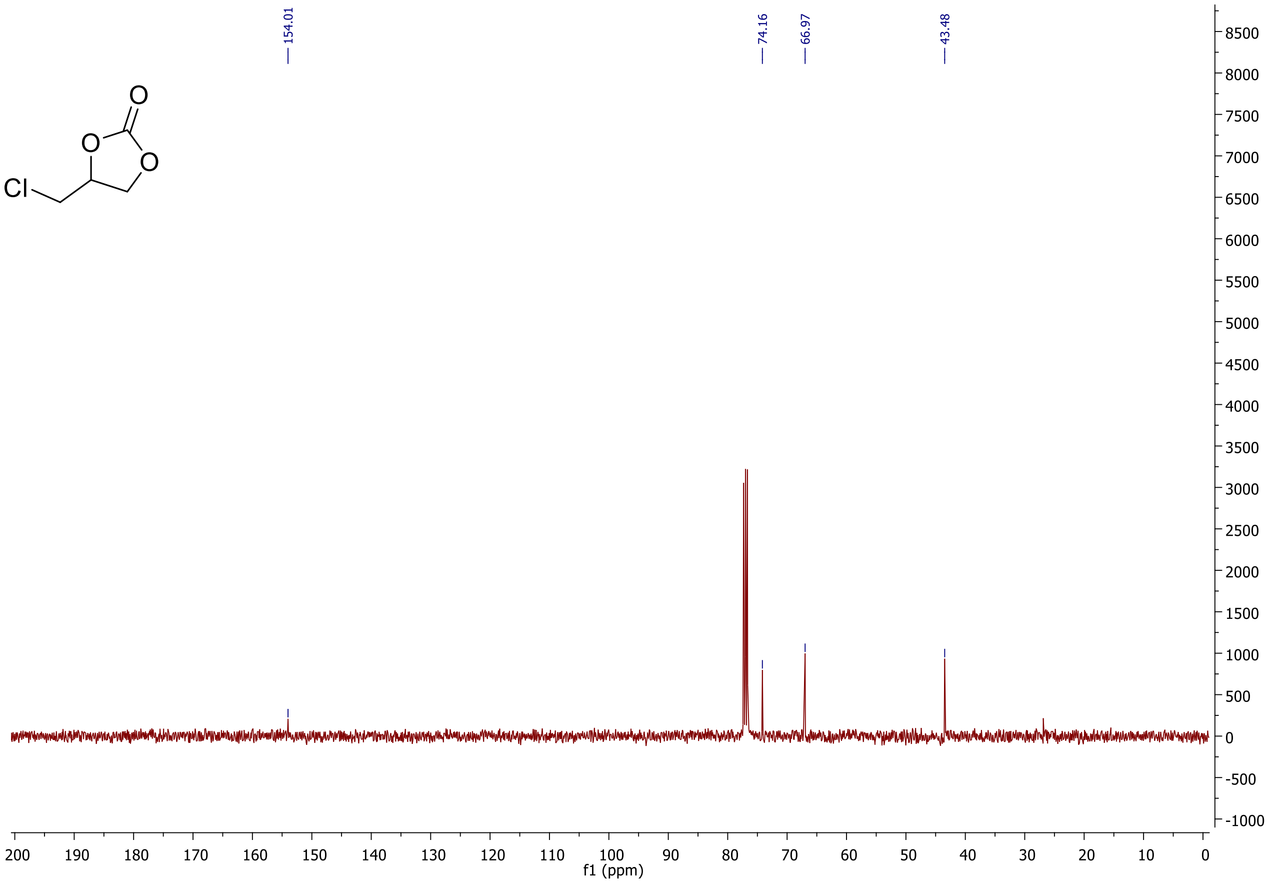

Figure S17. ^{1}H-NMR (300 MHz, $CDCl_3$), ^{13}C{^{1}H}-NMR (101 MHz, $CDCl_3$) of (**18f**).

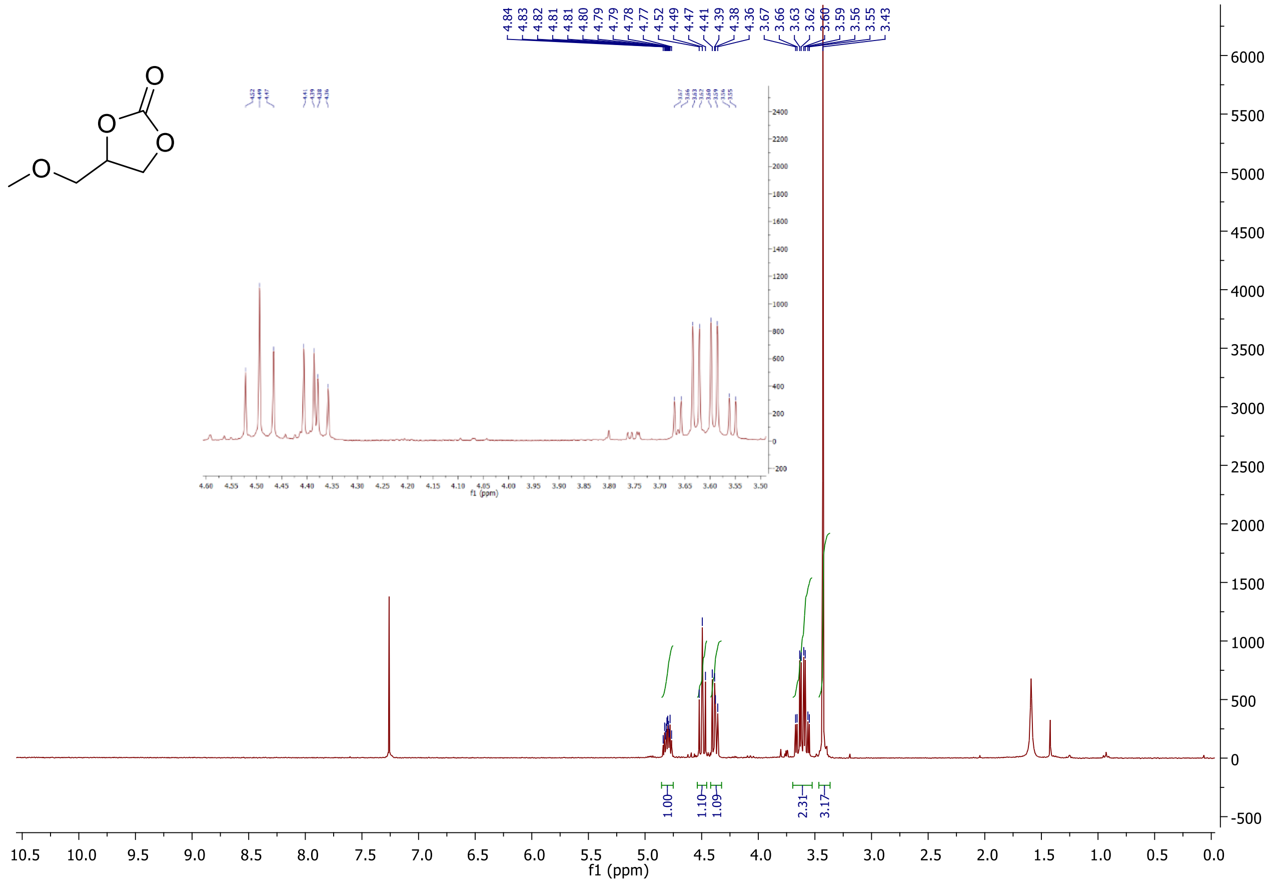

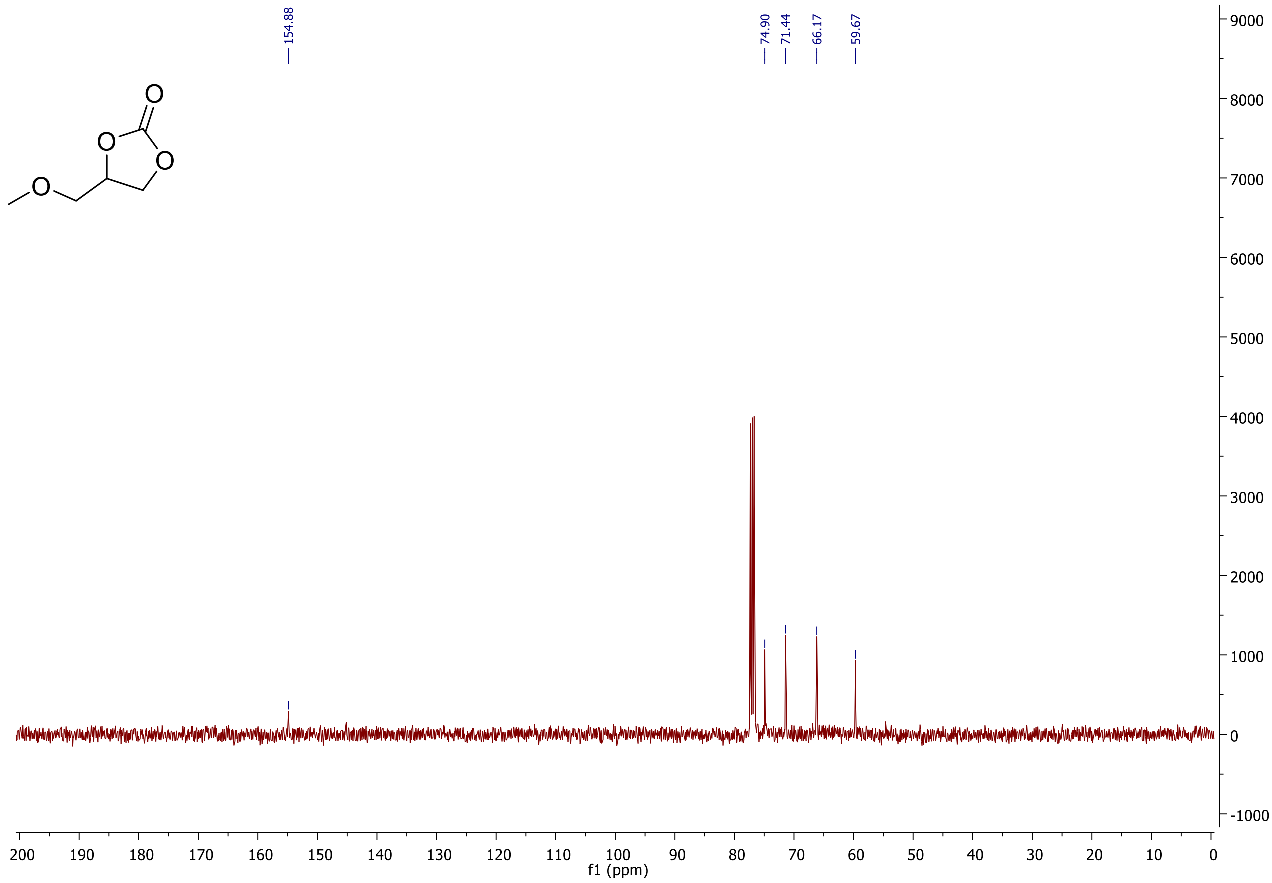

Figure S18. ^{1}**H-NMR (300 MHz, CDCl**$_3$**),** ^{13}**C{**^{1}**H}-NMR (101 MHz, CDCl**$_3$**) of (18g)**.

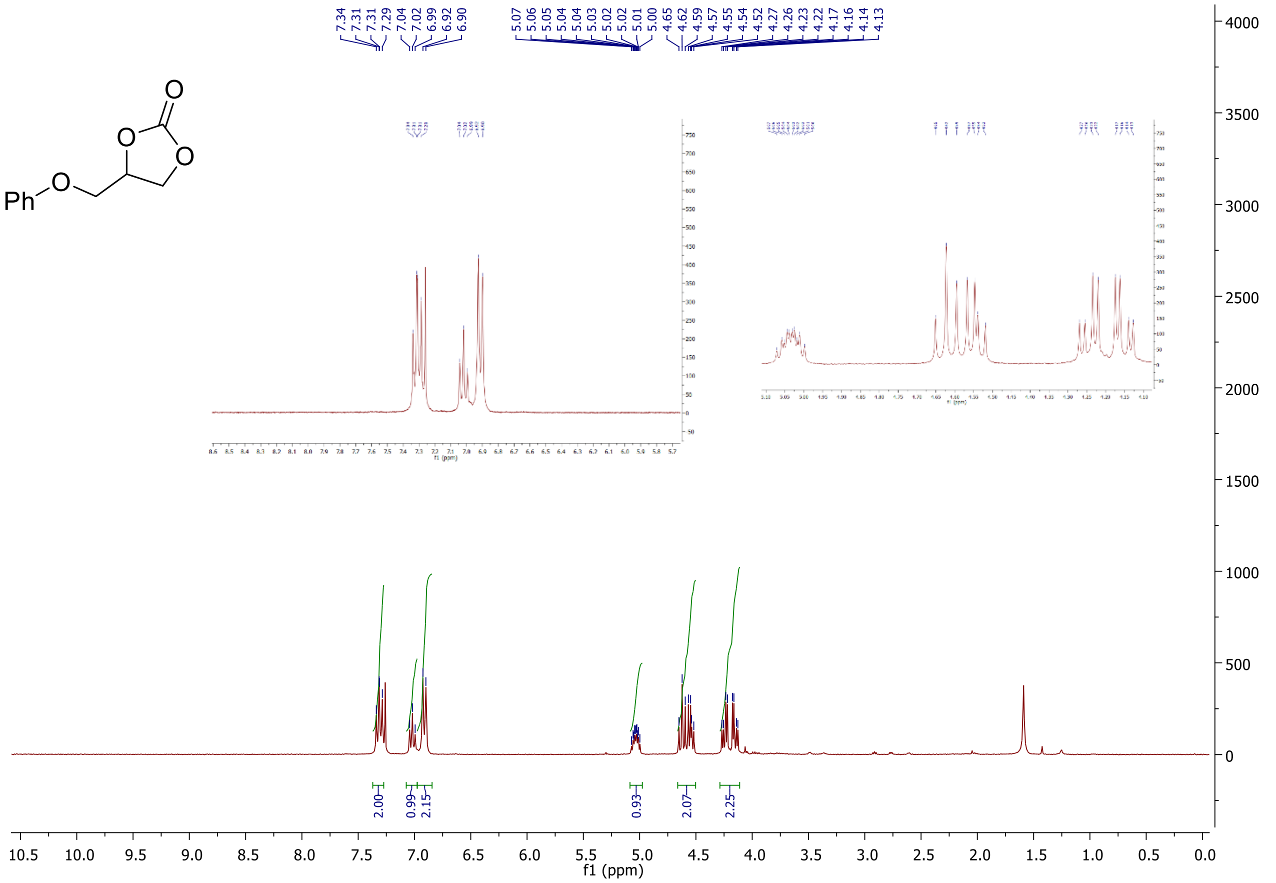

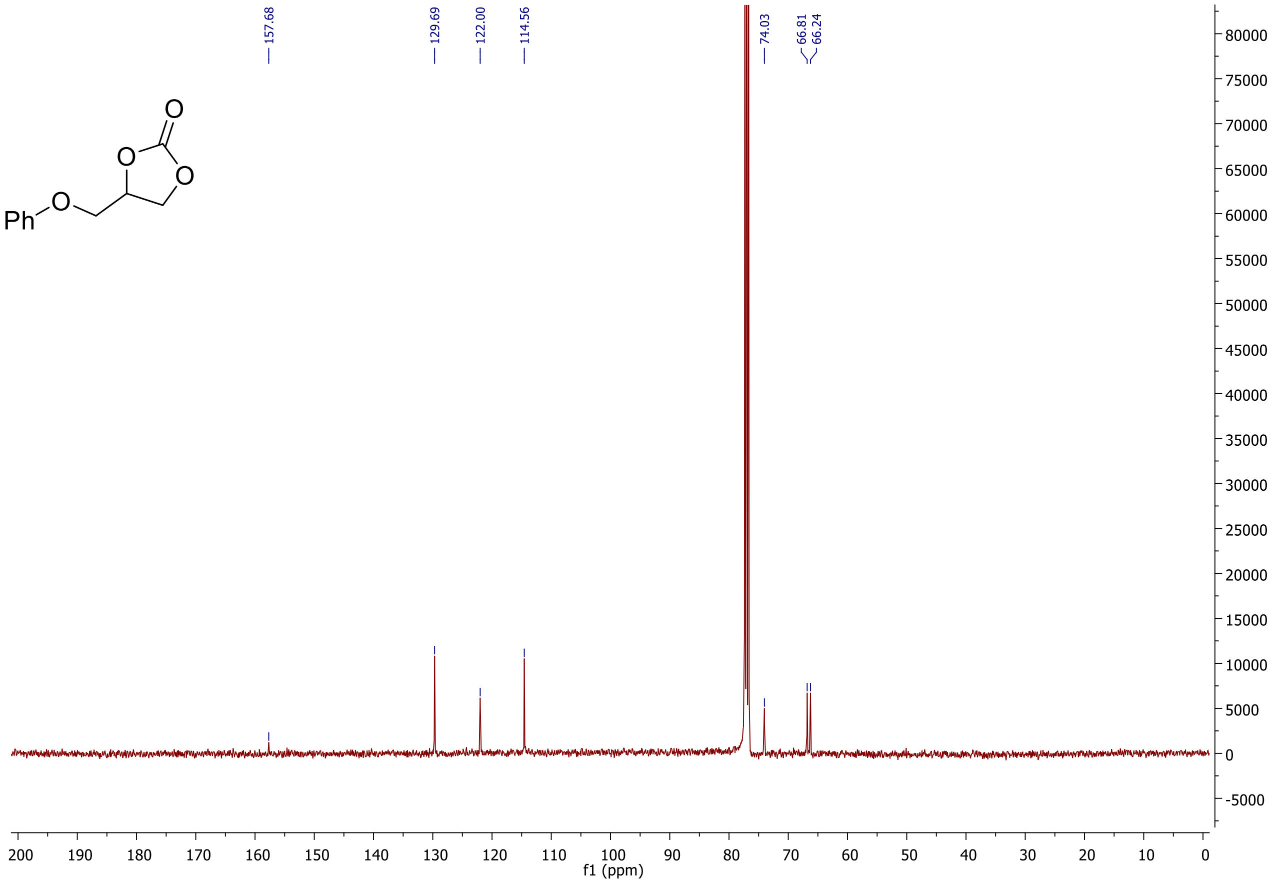

Supplement: Supplementary file 1 [file molecules-28-01530-s001.zip › molecules-2150696-supplementary.pdf]
